# Distribution and relative age of endemism across islands worldwide

**DOI:** 10.1038/s41598-019-47951-6

**Published:** 2019-08-12

**Authors:** Simon Veron, Thomas Haevermans, Rafaël Govaerts, Maud Mouchet, Roseli Pellens

**Affiliations:** 1 Institut de Systématique, Evolution, Biodiversité (ISYEB UMR 7205), Muséum National d’Histoire Naturelle (MNHN), CNRS, Sorbonne Université, EPHE, CP 51, 47 rue Buffon, 75005 Paris, France; 2 Centre d’Ecologie et des Sciences de la Conservation (CESCO UMR 7204) MNHN, CNRS, Sorbonne Université - CP51, 55–61 rue Buffon, 75005 Paris, France; 3 Royal Botanic Gardens, Kew, Richmond, Surrey, TW9 3AE UK

**Keywords:** Biogeography, Conservation biology

## Abstract

Islands have remarkable levels of endemism and contribute greatly to global biodiversity. Establishing the age of island endemics is important to gain insights into the processes that have shaped the biodiversity patterns of island biota. We investigated the relative age of monocots across islands worldwide, using different measures of phylogenetic endemism tested against null models. We compiled a species occurrence dataset across 4,306 islands, and identified 142 sites with neo-, paleo-, mixed and super-endemism. These sites were distributed across the world, although they tended to be more common at low latitudes. The most frequent types of endemism were mixed and super-endemism, which suggests that present-day island biodiversity has frequently been shaped by processes that took place at different points in times. We also identified the environmental factors that contributed most to different types of endemism; we found that latitude, habitat availability and climate stability had a significant impact on the persistence of ancient taxa and on recent diversification events. The islands identified here are irreplaceable both for the uniqueness and the evolutionary history of their flora, and because they are a source of “option values” and evolutionary potential. Therefore, our findings will help guide biodiversity conservation on a global scale.

## Introduction

Ever since Charles Darwin’s seminal studies on the Galápagos Archipelago, and those of Alfred Russel Wallace on the Malay Archipelago, island biogeography has made significant contributions to the foundations of modern ecology and evolutionary biology^1^. The isolation of life on islands has allowed evolution to take its own course, resulting in a multitude of unique lineages and communities found nowhere else on Earth^2^. Although the total number of species on islands is lower than on continents, the prevalence on islands of high endemism at all taxonomic levels means their contribution to global biodiversity is huge^3–6^. Most areas across the globe with exceptional rates of endemism are islands, e.g. New Caledonia, Hawaii, Madagascar, and islands in the Mediterranean region^7^. This extraordinary recurrence provides a unique opportunity to understand the diversification of life on Earth. Although there have been a number of studies documenting the processes leading to endemism on single islands or archipelagos^6,8–11^, little is known about the large scale processes underlying the origin and persistence over time of endemics (but see^5^).

One intriguing observation concerning endemism is that there are sites across the world with significant concentrations of organisms whose ages depart from the average, i.e. they are either younger or more ancient^12–15^. Paleo-endemism is associated with relicts of clades that used to be richer and have larger distributions. High concentration of paleo-endemics on a given site suggests that there may be particular conditions for the long-term survival of these species, which either diversified or disappeared elsewhere. Neo-endemics are recently diverged species, and sites with neo-endemics are assumed to provide conditions for bursts of diversification. Paleo- and neo-endemism have been associated with the origin of island biota^14^. A key assumption is that neo-endemism is associated with oceanic islands, i.e. islands formed *de novo*, whereas paleo-endemism is linked to continental islands, i.e. islands formed by fragmentation. Neo-endemism results from speciation after immigration, which, given enough time, could lead to adaptive radiations. Paleo-endemism is assumed to result from an opposite process called relictualization, corresponding to species survival and evolution on an island and extinction on the landmass from which they originated. Gillespie & Roderick^14^ also introduced the concept of mixed endemism to account for cases where both neo- and paleo-endemism are observed. However, despite these theoretical considerations and a number of enlightening examples, this problem has so far only been investigated in small clades within a localized or regional biogeographical framework (e.g.^12^). The extent to which this phenomenon can be generalized to islands across the world and to large groups of organisms has never been investigated and very little is known for corroborating or refuting the hypothesis associated to their origin. In addition, the insular abiotic factors that may explain the high concentration of neo-endemic and /or paleo-endemic species remain to be explored.

Recent advances in molecular sequencing have enabled the generation of comprehensive dated phylogenies of very diverse and widely distributed groups of organisms. These data are completely shifting the scale at which some studies can be conducted, allowing hypotheses to be tested on a more general and repeatable basis^16–18^. Combining occurrence data and phylogenies has been made possible to reveal the relative age of insular communities, in particular using measures of phylogenetic endemism^17,19–21^.

In this study, we take advantage of these recent developments to (1) test whether there are islands that harbor floras with significant neo-, paleo-, or mixed-endemism; (2) identify these islands and investigate the geographical, historical and environmental factors that may explain the observed patterns of endemism. We focused on monocots, a large group of plants distributed across the globe and well represented on islands. Monocots form a morphologically and functionally diverse clade representing a quarter of flowering plant diversity; they comprise for example orchids, palms and cereals, with many species of particular value for humanity, providing food and other resources. In addition, several species contribute significantly to primary production in open ecosystems.

## Results

### Spatial patterns of paleo-, neo-, mixed and super-endemism

**Figure 1:**
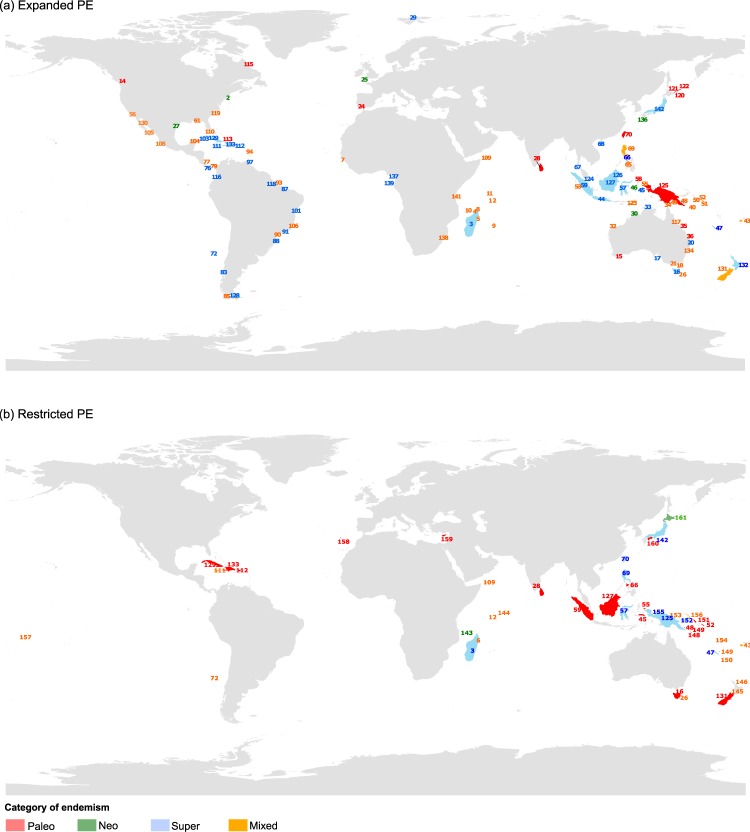
Spatial distribution of areas of paleo-, neo-, mixed and super-endemism. (**a**) All monocot genera found on islands are considered equally. (**b**) Monocot genera also found on continents are weighted lower. Numbers shown are ID numbers given to each island (see Supplementary Datasets S1 and S2); complete analysis results are given in Supplementary Datasets S1 and S2.

Of the 4,306 islands included in this study, only 142 (i.e. 3.3% of all islands analyzed) were found to belong to one of the four categories of endemism based on the Expanded Phylogenetic Endemism index (PE_E_). Expanded Phylogenetic Endemism measures the geographical concentration of evolutionary history of all species native of islands, i.e. that can also be present on continents (see Methods). Note that phyologenetic endemism departs from the traditional definition of endemism as it does not represent the confinement of a species to a discrete geographic unit but measures the geographical concentration of the evolutionary history of a set of species compared to a broad landscape where diversity is distributed to varying degrees^17,19,20^. We found that 16 islands (0.37%) were identified as areas of expanded paleo-endemism, 10 (0.23%) were areas of expanded neo-endemism, 74 (1.7%) were areas of expanded mixed endemism, and 42 (0.97%) were areas of expanded super-endemism. Super-endemism is a sub-category and an extreme case of of mixed endemism (see Methods). Islands with significant PE_E_ values were found across the globe but were more frequent in the Southern Hemisphere (Fig. 1a, Table 1). Significant areas of endemism were predominantly of the super- (e.g. Madagascar, Borneo, Sumatra, New Caledonia, North Island in New Zealand, Cuba) and mixed endemism type (e. g. Luzon in the Philippine archipelago, Timor in Indonesia, South Island in New Zealand, Dominica in the Caribbean). Areas of paleo-endemism were found in the Indian Ocean (Sri Lanka), Australia (e.g. Bald Island, Henning Island), Taiwan, New Guinea, on islands north of Japan (Iturup Island, Kunashiri Island, Shikotan Island), and south of Spain (Isla Saltés). Some of the few islands with neo-endemism include Isla Chepillo, Brava Island, Montuosa Island (all in Panama), Ilha Furtada in Brazil, and Kuro Island in Japan.

**Table 1:** Estimates, standard errors (SE) and weights (Σ*w*_*i*_) for each abiotic factor calculated from multimodel selection using (a) PE_E,_ expanded insular phylogenetic endemism, and (b) PE_R_ restricted insular phylogenetic endemism.

		Significant endemism			Paleo-endemism			Neo-endemism			Mixed endemism			Super- endemism		
		Estimate	SE	Σ*w*_*i*_	Estimate	SE	Σ*w*_*i*_	Estimate	SE	Σ*w*_*i*_	Estimate	SE	Σ*w*_*i*_	Estimate	SE	Σ*w*_*i*_
**(a) Expanded insular phylogenetic endemism (PE**_**E**_ **)**																
Localization	Minimum distance from the continent	**−0**.**27**	**0**.**10**	**0**.**98**	0.08	0.31	0.26	−0.84	0.81	0.41	−0.06	0.17	0.28	**−0**.**29**	**0**.**10**	**0**.**99**
	SLMP	−0.16	0.12	0.48	−0.18	0.49	0.30	0.18	0.63	0.28	**0**.**53**	**0**.**19**	**0**.**96**	**0**.**20**	**0**.**11**	**0**.**64**
	Latitude	**−1**.**7**	**0**.**12**	**1**	0.44	0.46	0.40	−0.22	0.96	0.32	**−1**.**59**	**0**.**22**	**1**	**−1**.**26**	**0**.**13**	**1**
	Longitude	**−0**.**5**	**0**.**09**	**1**	**0**.**68**	**0**.**38**	**0**.**75**	−0.42	0.41	0.38	**−0**.**36**	**0**.**10**	**0**.**99**	**−0**.**25**	**0**.**07**	**1**
Habitat availability	Elevation	**0**.**17**	**0**.**09**	**0**.**72**	**0**.**51**	**0**.**17**	**0**.**93**	−0.008	0.60	0.26	**0**.**25**	**0**.**11**	**0**.**85**	−0.01	0.08	0.28
	Area	0.07	0.14	0.30	−0.14	0.18	0.36	−6.77	23.14	0.29	−0.13	0.09	0.32	−0.07	0.06	0.43
	Number of ecoregions	−0.11	0.08	0.48	0.18	0.13	0.58	−7.35	815.83	0.35	**0**.**40**	**0**.**08**	**1**	0.009	0.06	0.27
Bioclimatic variables	Mean annual temperature	**0**.**79**	**0**.**15**	**1**	−0.22	0.40	0.31	1.56	0.96	0.71	**0**.**89**	**0**.**35**	**0**.**92**	**0**.**67**	**0**.**18**	**1**
	Mean annual rainfall	0.13	0.08	0.56	−0.33	0.34	0.37	0.67	0.41	0.56	**0**.**30**	**0**.**14**	**0**.**76**	**0**.**31**	**0**.**09**	**1**
	Rainfall seasonality	0.068	0.12	0.32	−0.01	0.33	0.36	0.028	0.48	0.30	**−0**.**39**	**0**.**17**	**0**.**86**	−0.09	0.09	0.41
	Mean annual wind speed	**−0**.**94**	**0**.**12**	**1**	−0.21	0.35	0.31	0.18	0.76	0.29	**−0**.**73**	**0**.**24**	**0**.**99**	**−0**.**55**	**0**.**14**	**1**
	Sd of vapor pressure	**0**.**46**	**0**.**08**	**1**	0.13	0.27	0.29	**0**.**75**	**0**.**30**	**0**.**91**	**0**.**23**	**0**.**14**	**0**.**61**	**0**.**25**	**0**.**07**	**1**
Past climate	Velocity of past climate change	**−0**.**17**	**0**.**10**	**0**.**61**	−0.95	0.83	0.52	0.22	0.75	0.27	−0.37	0.55	0.32	−0.14	0.20	0.32
	GMMC 1 = connexion to continent during the last glacial maximum	−0.07	0.22	0.29	0.51	0.72	0.32	−0.09	1.10	0.28	0.04	0.36	0.28	−0.029	0.22	0.30
Sampling effort	ICEr	**−0**.**61**	**0**.**085**	**1**	−0.13	0.31	0.28	−0.29	0.53	0.29	**0**.**52**	**0**.**14**	**1**	**−0**.**29**	**0**.**086**	**1**
(**b**) **Restricted insular phylogenetic endemism** (**PE**_**R**_)																
Localization	Distance to the closest continental area	0.17	0.21	0.33	−0.28	0.46	0.31	**−8**.**7**	**4**.**9**	**1**	0.18	0.25	0.31			
	SLMP	−0.24	0.39	0.33	−0.57	0.53	0.43	−0.27	0.99	0.28	−0.32	0.51	0.34			
	Latitude	**−0**.**74**	**0**.**32**	**0**.**89**	0.26	0.59	0.31	−0.59	1.1	0.32	**−1**.**08**	**0**.**44**	**0**.**92**			
	Longitude	**0**.**59**	**0**.**23**	**0**.**97**	**0**.**61**	**0**.**35**	**0**.**71**	0.73	0.78	0.38	0.36	0.29	0.49			
Habitat availability	Area	−0.028	0.089	0.28	**−0**.**38**	**0**.**17**	**0**.**97**	−0.05	0.68	0.30	0.066	0.12	0.31			
	Elevation	**0**.**36**	**0**.**1**	**1**	**0**.**63**	**0**.**17**	**1**	0.24	0.33	0.32	−0.0065	0.18	0.27			
	Number of ecoregions	**0**.**59**	**0**.**11**	**1**	**0**.**33**	**0**.**15**	**0**.**95**	0.27	0.25	0.38	**0**.**27**	**0**.**10**	**0**.**96**			
Bioclimatic variables	Mean annual temperature	0.45	0.42	0.44	**1**.**19**	**0**.**7**	**0**.**69**	−0.74	1.07	0.32	0.43	0.58	0.37			
	Mean annual rainfall	0.26	0.20	0.46	0 0.11	0.38	0.31	−0.1	0.81	0.27	0.22	0.28	0.34			
	Rainfall seasonality	−0.037	0.24	0.28	−0.62	0.51	0.31	0.63	0.64	0.37	−0.14	0.31	0.30			
	Mean annual wind speed	**−0**.**58**	**0**.**26**	**0**.**82**	−0.76	0.49	0.59	−1.09	1.01	0.43	**−0**.**71**	**0**.**38**	**0**.**78**			
	Sd of vapor pressure	0.13	0.20	0.42	−0.27	0.36	0.35	0.66	0.56	0.55	0.26	0.26	0.37			
Past climate	Velocity of past climate change	−1.37	1.46	0.41	−1.9	2.3	0.39	0.63	1.25	0.29	−1.9	2.3	0.38			
	GMMC 1 = connexion to continent during the last glacial maximum	**−1**.**35**	**0**.**47**	**0**.**96**	0.13	0.74	0.28	−1.2	1.9	0.32	**−1**.**6**	**0**.**7**	**0**.**92**			
Sampling effort	ICEr	**0**.**44**	**0**.**20**	**0**.**83**	0.49	0.33	0.55	0.16	0.71	0.27	**0**.**51**	**0**.**29**	**0**.**70**			

Bold values indicate statistical significance (p-value < 0.001). Explanation of abreviations can be found in Table 3.

**Figure 2:**
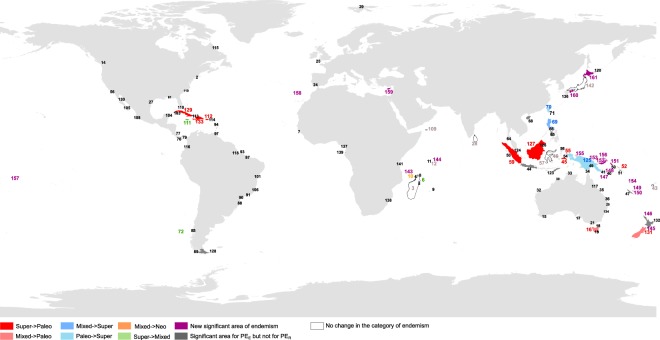
Map of endemism category changes between expanded PE and restricted PE. The arrows in the legend show the direction of change. Numbers shown are ID numbers given to each island; analysis summary is given in Supplementary Dataset S3.

By comparison, 47 significant areas of endemism were identified using the Restricted Phylogenetic Endemism index (PE_R_): 20 islands (0.46%) with paleo-endemism, 3 (0.07%) with neo-endemism, 16 (0.37%) with mixed-endemism, 8 (0.18%) with super-endemism. Restricted Phylogenetic Endemism is similar to Expanded Phylogenetic Endemism but is restricted to native species found on islands only and with no recorded occurrence on the mainland. Most of the islands identified using the PE_R_ index were found in the same category as with the PE_E_ index, with a few exceptions. In particular, additional paleo-endemic areas were identified using PE_R_ (Fig. 2; e.g. Cuba, Cyprus, New-Zealand). In the Indonesian islands, paleo-endemism (e.g. Borneo, Sumatra) and super-endemism (e.g. Sulawesi, New Guinea) were more frequent with PE_R_ than with PE_E_. Conversely, although the PE_E_ index identified islands of South and Central America as having predominantly mixed and super-endemism, these were no longer identified as significant areas of endemism with the PE_R_ index. The islands of the West Indies were one exception, with a shift from super- (PE_E_) to paleo-endemism (PE_R_) (Fig. 2; Cuba, Hispaniola, Puerto Rico). Likewise, Australian islands were no longer identified as areas of significant endemism with the PE_R_ index, with the exception of Tasmania (paleo-endemism) and Sisters Island of Tasmania (mixed-endemism). Only three islands were found to be areas of significant neo-endemism: Mayotte, Hokkaido and Anjouan.

### Predictors of phylogenetic endemism

**Figure 3:**
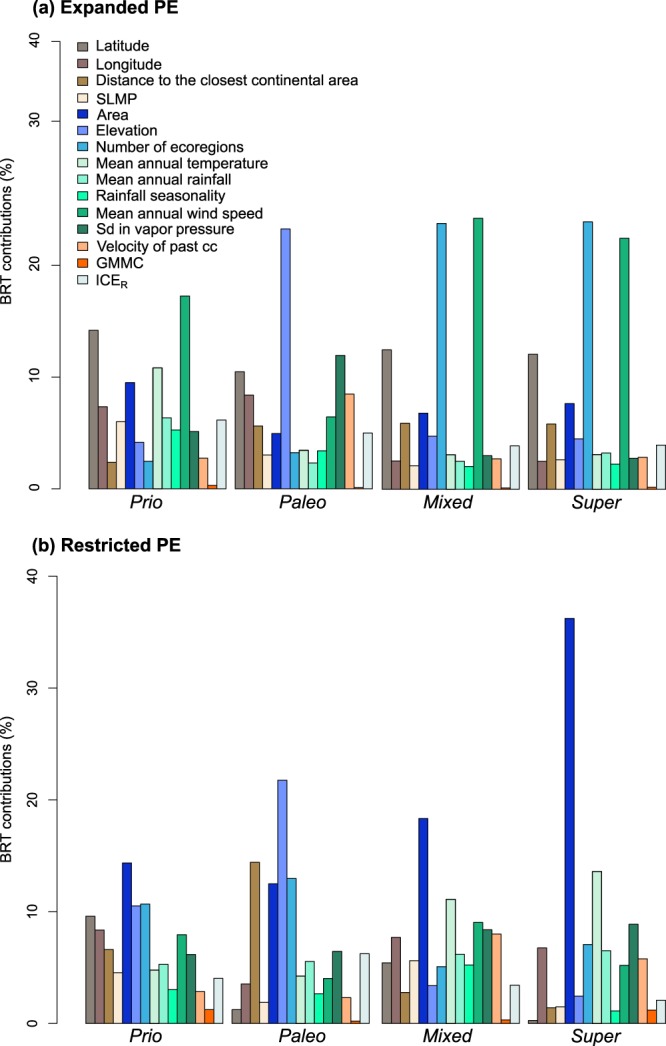
Contributions of abiotic variables to categories of endemism estimated from Boosted Regression Trees. Form of the relationship is provided in Supplementary Fig. S1.

Boosted Regression Trees and multi-model selection methods indicated that the factors that contributed the most (c%) and that had a significant effect on areas of endemism identified with PE_E_ were temperature (c = 10.8%; positive effect), latitude (c = 14.3%; negative effect), area (c = 9.5%; positive effect) and wind speed (c = 17.1%; negative effect) (Fig. 3; Supplementary Fig. S1). However, predictors of expanded endemism generally differed between categories (Fig. 3, Table 1). The variables that contributed most to paleo-endemism were elevation (c = 23.2%; significant positive effect), standard deviation in vapor pressure (c = 11.9%; no significant effect), and latitude (c = 10.4%; no significant effect). For islands with mixed and super-endemism, several variables were highly weighted in the process of multi-model selection and had a significant effect, in particular latitude (significant negative effect), longitude (significant negative effect), mean annual wind speed (significant negative effect), the number of ecoregions (significant positive effect), and mean annual rainfall (significant positive effect). Variables with the greatest contribution, estimated from BRT, were mean annual wind speed (c = 24.2%, 22.5% for mixed and super-endemism, respectively) and the number of ecoregions (c = 23.7%, 23.9% for mixed and super-endemism, respectively) (Fig. 3; Supplementary Fig. S1). Due to the small number of islands in the neo-endemism category, the BRT algorithm did not converge. However, multi-model selection indicated a slight positive significant effect of standard deviation in vapor pressure.

For areas of significant endemism identified with the PE_R_ index, all categories combined, the factors with the highest contributions were elevation (c = 10.5%), longitude (c = 8.3%), the number of ecoregions per island (c = 10.6%), and sampling effort (c = 4.03%), which all had positive effects. Latitude (c = 9.5%) and wind speed (c = 9.5%) also made a relatively high contribution but had a negative effect. When assessing the contribution and the effects of abiotic variables on categories of endemism separately, we found that the BRT algorithm did not converge when testing the contributions to neo-endemism and that the multi-model algorithm did not converge for super-endemism. Island area made a high contribution to all categories of endemism. However, for islands with paleo-endemism, BRT fitted functions showed a positive effect of “island area” whereas multi-model selection indicated a significant negative effect. To account for the potential limitations in our model (especially residual collinearity), we performed a generalized linear model test with “island area” as the unique response variable. This indicated that island area had a positive effect on paleo-endemism, similar to the results found with the BRT algorithms. Furthermore, the number of ecoregions was found to have a significant positive effect on islands with paleo- and mixed endemism, although its relative contribution was higher for the former (Fig. 3; Supplementary Fig. S1). Among predictors that had a significant effect or made a high contribution to a single category of endemism (concerning only paleo- and mixed-endemism), we found that the distance from the nearest continental area made a high contribution to paleo-endemism (but its effect was not significant) and that longitude and mean annual temperature had slightly significant positive effects. Regarding mixed-endemism, we found that past connection to the continent and latitude had significant negative effects.

## Discussion

Although both the importance of island endemism for global biodiversity and the value of phylogeny for understanding the diversification and distribution of life on Earth are well-recognized, the integration of phylogeny in the study of endemism on a macroscale is only just beginning^17,21,22^. Our study shows how this approach can help identify endemism hotspots worldwide, using monocots as an example. We were able to identify islands from all over the world that have significant levels of endemism and determine the origin of this endemism based on four relative age categories.

The relatively high number of islands in the mixed and super-endemism categories (e.g. Madagascar, Mayotte, Luzon, Dominica) is one of the main findings of this study. As these categories are defined by the co-occurrence of short and long branches that are rare on the landscape^21^, this result highlights that co-occurrence of groups of contrasting age within an island is relatively common worldwide. This pattern has been well-documented in New Caledonia. As shown by Nattier *et al*.^23^, the bulk of New Caledonia’s biota is quite recent, even though it is the oldest oceanic island in the world^24,25^. However, the island also harbors some endemic species found on very long branches, indicating they are the present survivors of clades that are much older than the island^16^. Examples include *Amborella trichopoda* the iconic monospecific genus that is sister to all extant flowering plants^26^, taxa belonging to the order Opliones (Arachnida)^27^, or the emblematic flightless Kagu bird (*Rhynochetos jubatus*)^28^. The islands identified here with mixed or super- monocot endemism may therefore be new places to study the co-occurrence of ancient and young taxa. They could be case studies to investigate the role of island and archipelago dynamics and their intricate contribution to long-term survival and evolution of species.

By contrast, very few islands were found to be significant centers of neo-endemism (e.g. some islands in Brazil, Panama, Indonesia), irrespective of which index was used. At first glance, the fact that our analysis was carried out at the genus level could explain this result, suggesting a distribution pattern where recent genera are rarely restricted to a few islands, whereas species could be. This pattern is known for Darwin’s finches in the Galápagos, with one species per island^2^, for spiders in Hawaii^29^, and Anolis lizards in the Carribean^30,31^ but has not really been documented for monocots. Future studies using phylogenies that include a representative sample of insular species will help determine the extent to which taxonomic level influences these results. Nevertheless, Mishler *et al*.^21^ also found a very low number of areas of neo-endemism for the genus *Acacia* in Australia. This suggests that neo-endemism may be less frequent than other categories regardless of the taxonomic level considered. In any case, it implies that genera on short branches (i) are either rarely found on only one or very few islands or (ii) rarely occur alone: as shown above, when rare short branches and rare long branches co-occur, the island is classified as an area of mixed or super-endemism. The fact that in this study, islands with neo-endemism are nearly all continental (based on both PE_E_ and PE_R_ indices) is another novel finding from this study. The ones identified with the PE_R_ index were connected to the continent until very recently (until the Last Glacial Maximum, i.e. 26–19 Ky BP), suggesting that differentiation giving rise to new genera may have happened over a very short time frame (e.g.^32^). Whittaker and Fernández-Palacios^4^ emphasized that recent speciation events have not occurred only on oceanic islands but also on continental islands. Another possibility is that taxa belonging to these relatively young genera went extinct on the continent and only those on islands survived, which would account for their apparent range-restriction^13,16^. Although more research is needed, this would go against the traditional way of viewing neo-endemics, which are often associated with oceanic islands and considered to be peripheral isolates that have evolved by *in-situ* speciation^14,21^.

Areas of insular paleo-endemism were found all over the world, and, contrary to theoretical expectations, on islands with contrasting histories. Paleo-endemics are expected to be found on islands produced via fragmentation of the continental crust and are thought to have arisen through relictualization^14^. However, extinctions on the continent may also have caused the evolutionary isolation of taxa found on oceanic islands, giving birth to species considered as paleo-endemics. Madeira, an island of oceanic origin, provided climatic refugia for tree species that went extinct on the mainland because of extreme climate changes^4^. Although many tree species currently present on Madeira are different from those in the fossil record, they are the last remnants of ancient lineages and are considered to be paleo-endemics^4^. Despite the fact that paleo-endemic lineages are ancient and range-restricted, they are found on islands that may be either ancient (South Island in New Zealand) or recent (Sri Lanka) in geological terms. The presence of paleo-endemic lineages on ancient islands may suggest these have been present since the islands’s origin (but see^33^), and that they have persisted because of the maintenance of suitable habitats and a lack of competitors, and/or predators. In addition, as mentioned above, some young islands are also areas of paleo-endemism. One possible explanation is that these paleo-endemics are relicts of a clade that colonized different islands of an archipelago, moving from one island to another as the one they lived on degraded and subsided^23,34^. This hypothesis could be tested with geological data showing whether this scenario could have occurred in regions with high paleo-endemism. Other types of dispersal events cannot be discounted. For example, ancient lineages may have dispersed from continents where they could have become extinct. Fossil records documenting the presence of similar or closely related clades on continents could confirm this kind of event. Therefore, an interesting line for future research would be to combine the effects of age and connection to the mainland and explore for which paleo-endemic island the “relictualization” or “dispersal” hypothesis is the most likely. Tasmania is a nice example of how geological history and island age estimate may influence phylogenetic endemism. Tasmania’s biota date back to Gondwana (i.e. about 80 My BP), although the island was only recently separated from Australia^35^. Consequently, Tasmania was classified as an area of paleo-endemism despite its relatively recent isolation. This is a well-known example, however, for many islands, information on the geological age is lacking. This highlights the need to synthesize the different parameters that are used to estimate island age and facilitate their integration in a database.

In this study, certain islands were found to belong to different categories depending on whether we considered expanded or restriced phylogenetic endemism (PE_E_ and PE_R_); this may be significant for understanding the “relictualization” process. The category that was most liable to vary between indices was “paleo-endemism”, with the identification of new areas such as Borneo, Sumatra, and certain Caribbean islands (Cuba, Hispaniola, Puerto-Rico). A change in the endemism category may reflect the difference in relative age between genera restricted to islands and those also found on continents. For example, a change from mixed-endemism to paleo-endemism (e.g. Tasmania) may suggest that island endemics could be relatively older than genera that are also found on the mainland, favoring the relictualization hypothesis. Moreover, a relatively high proportion of islands did not change category between expanded and restricted phylogenetic endemism. This is the case for most of islands identified as significant areas of restricted endemism, in particular those situated in the Indian Ocean (e.g. Madagascar, Sri Lanka, Socotra). An island may be identified as belonging to the same category with either index because even when continental genera are included, the global age of the community is determined by the age of genera that are only found on islands. Another explanation may be that, on those islands, endemic genera that are restricted to islands and those that are also found on the mainland generally have a similar age.

Latitude was the localization variable that contributed the most to nearly all categories of endemism, for both restricted and expanded phylogenetic endemism. This is congruent with global patterns of biodiversity distribution, where higher richness and endemism are observed at lower latitudes for most taxonomic groups^5,36^. The fact that latitude is a significant factor for all categories of endemism suggests that different and non-exclusive processes may underlie the higher diversity observed in the tropics (as already indicated by Gaston^37^), and that, for monocots at least, the tropics may be both a cradle, i.e. the source of new species, and a museum, i.e. where old species persist, of diversity^36^. The high availability of energy in the tropics, as reflected by the contribution of vapor pressure and temperature to endemism rates, may allow the co-occurrence of range-restricted species resulting in high phylogenetic endemism^17,38^. At low latitudes, temperatures are high nearly all year round, with much greater diurnal than seasonal variations. These diurnal variations are known to increase rock degradation, soil creation, organic matter decomposition and nutrient cycling, all of which are factors that are crucial for the establishment of plants. In addition, high mean temperatures imply lower environmental selection on organismal life cycles, so that annual, biannual, and perennial organisms may co-occur. Therefore, the high contribution of mean annual temperature to islands classified as areas of mixed and super-endemism (with the PE_R_ index) probably highlights the influence of this environmental factor on the establishment and diversification of plants on islands^39^.

Wind speed was found to have a significant negative effect on the occurrence of areas of mixed and super-endemism, which may reflect the substantial contribution of wind on dispersal filtering rather than on environmental filtering. Indeed it would be expected that frequent strong winds may act as a climatic barrier on island’s flora and select forms adapted to desiccation and rapid germination. Island floras that are exposed to strong winds, frequent storms and cyclones would be expected to be mainly composed of organisms capable of withstanding these harsh conditions. However, our results suggest that strong winds may on the contrary be a force selecting for organisms that tend to be more ubiquitous and able to colonize islands multiple times. Low wind speed, on the other hand reduces the role of wind for dispersal, thereby contributing to endemism. Similarly, island remoteness may act as a dispersal filter, and offer conditions for species survival and diversification, promoting phylogenetic endemism and in particular paleo-endemism^14^. Low wind speed is also a factor that decreases the stress on island floras and may be related to climatic stability. Climatic stability was shown to be very important for the persistence of old clades as well as for promoting diversification, as found for continental faunas^40^. Notably, we observed that slow velocity of past climate change correlates with significant phylogenetic endemism. These results highlight the importance of integrating wind speed, direction and variation in studies of island biogeography. It also shows the need to integrate phylogenetic information with trait diversity, which could be done for monocots, in order to assess the way wind dispersal and island isolation may select plant species and lead to island diversity.

Finally, habitat availability had a key importance to explain phylogenetic endemism. Area contributed highly to all categories of endemism (e. g. to paleo-endemism in Sumatra and Borneo, to super-endemism in Madagascar and New-Guinea), suggesting it is crucial for taxon’s establishment and long-term persistence, and for giving rise to uniqueness within the world’s flora. Indeed, large islands not only offer the possibility of large population sizes, they also have diverse environments forming geographical barriers that could lead to allopatric speciation *in situ*^2^ which, over time, could ultimately lead to single island endemics (see the model from Whittaker *et al*.^34^). Corroborating this assumption, the number of ecoregions also contributed highly to age categories, suggesting the importance of habitat availability for the co-occurrence of rare short and long branches^41^. Finally, elevation was also found to be an important contributor for nearly all categories of endemism. This result highlights the importance of mountains, which support diverse habitats resulting from altitudinal gradients, different climatic conditions between windward and leeward sites, and high precipitation regimes in certain altitudinal belts. In addition, elevation has played an important role in providing refuges of suitable habitats during the climatic fluctuations of the Pliocene and Pleistocene. For islands near the tropics, particularly in the Southern Hemisphere, elevation has contributed to the maintenance of humid forests during glacial periods when places at low altitudes became much drier^42^. In particular, the high contribution of elevation to paleo-endemism, for both expanded and restricted phylogenetic endemism, is consistent with the idea that these refuges played a key role in the survival of ancient lineages^43^. It is conceivable that on some islands that became periodically dry, mountains offered more suitable conditions for these lineages.

We cannot exclude that the data used here contained certain biases that are inherent to natural history collections records^44^. These include the fact that places that are rich in biodiversity, easily accessible, close to academic structures, or of economic importance will tend to be better sampled (e.g.^45^). Therefore, it is likely that remote islands with low economic or biodiversity attractiveness and with no links to academically developed countries (currently or in the past) will be less sampled. Another bias is the human tendency to pay more attention to rare organisms^46^. Although this tendency would increase the diversity estimate of a region, by encouraging the sampling of rare and endemic taxa, it could impact our analysis by inflating estimates of endemism.

Nevertheless, as most of the largest herbaria of the world are nearly completely digitized^47^ the number of available recorded occurrences is huge (we used more than 2 million). We performed several procedures to reduce potential biases (i.e. crossing occurrences with the eMonocot database, using an index of geographic coverage to account for spatial gaps). In addition, considering endemism at the genus rather than the species level is also likely to lessen this bias: when a collector visits an island he/she is more likely to come across all the genera rather than all the species present. Therefore, we predict that future field surveys and further investigation of island floras are unlikely to change the categories identified here. They may, however, lead to the identification of additional islands with significant phylogenetic endemism.

To conclude, this study allowed to identify islands across the world with significant phylogenetic endemism for monocots. As phylogenetic endemism captures how much of the tree of life is restricted to a single or to a few places worldwide, these results highlight the irreplaceable quality of these islands (especially those detected with PE_R_ index), and the need for conservation in order to avoid the loss of deep branches in the tree of life^17,20,48^. In general, the islands identified here are already areas of top priority for conservation on the basis of their species richness, level of endemism or because they are under threat (e.g. Madagascar, New-Guinea, New Caledonia, the Caribbean Islands)^5,7,17^. By conducting our study on a large number of islands worldwide and by including phylogenetic information, we were able to identify potentially undetected key areas for conservation (e.g. islands in Australia, South America, and Japan).

The rationale for using phylogenetic diversity as a central measure for biodiversity conservation is well known^49–52^. Based on the assumption that shared traits are due to a shared evolutionary history, phylogenetic diversity measures the variety of features produced by life. It is also considered the best way to assess the variety of options that could allow adaptation to a changing environment and provide benefits for future human needs^49,50^. These “option-values” can be considered “a safety net of biological diversity for responding to unpredictable events or needs (…)”^49^. Our approach based on phylogenetic endemism may therefore identify islands that may be irreplaceable for, as stated above, the preservation of deep branches of the tree of life but also of option-values. Monocots are a particularly important group regarding option-values, when we consider all the benefits they already provide for humanity.

A final important point is that we are currently unable to predict the way biodiversity will evolve, and which lineages (ancient or young) will be favored^51^. If we take, for example, the flowering plant *Amborella* or the lizard-like reptile *Tuatara*, we realize that their rates of molecular evolution are quite high, despite being found on long deep branches^16,18^, thus contradicting the *a priori* assumptions that ancient lineages are evolutionary dead-ends and that young lineages have a greater capacity to diversify and adapt. All that we know is that increased phylogenetic diversity will enhance the probability of having the right feature at the right time^52^, which leads us to expect that phylogenetic diversity might represent a good marker of evolutionary potential^50^. By preserving areas of significantly high PE we may increase the probability of having species that can contribute to the evolutionary potential of a clade.

## Methods

### Phylogeny

In this study, we used the monocot phylogeny of Tang *et al*.^53^, a dated phylogeny with 20 fossil and/or molecular calibration points. It is the most comprehensive monocot phylogeny to date and is well resolved for nearly 70% of the 2,823 recognized genera.

### Plant occurrences

Plant occurrence points were downloaded from the Global Biodiversity Information Facility (GBIF) portal (www.gbif.org), and cross-checked in the eMonocot database (http://e-monocot.org/). We extracted all records, except fossil specimens, from the GBIF portal and selected monocot taxa by using all names found in the eMonocot database as queries. The eMonocot database provides a comprehensive list of synonyms for the great majority of species, distribution verified by experts within polygons at the TDWG level 4 scale^54^, and native or non-native species status. Both the GBIF and eMonocot are continuously updated and cover a time span of at least 500 years. We used the eMonocot database to (*i*) check for synonymy (*ii*) verify the native and non-native status of GBIF occurrences, and (*iii*) check for discrepancies in distribution records, and we consequently excluded data from GBIF that deviated inexplicably from the eMonocot data.

Regarding (*ii*), we aimed to keep only native genera (Supplementary Fig. S2) and proceeded as follows. First, we compared native genera found in the eMonocot database with the occurrence data extracted from GBIF. As different spatial scales for islands and polygons were used in eMonocot and GBIF, this procedure was only valid for a small set of islands. For these islands, we kept only genera that were present in the eMonocot database and identified as native (Supplementary Fig. S2). All other islands were associated with a higher scale polygon defined in the eMonocot database, i.e. TDWG4 polygons. When a genus was identified as non-native to this polygon, the occurrence was discarded. However, as information about native and non-native status was sometimes missing in the eMonocot database, we employed a second test to identify genera occurrences found outside their native range (referring to points (*ii*) and (*iii*)). This was done by building polygons delimited by the maximal and minimal latitude and longitude of their known native ranges and discarding all occurrences outside these limits.

### Islands

We used the Global Island Database provided by the United Nations Environment Programme^55^, which comprises information on 180,495 islands worldwide, as the basis of our island dataset. We considered islands to be isolated areas surrounded by water and smaller than Australia; we included only continental and oceanic islands, and excluded those found within continents (e.g. in lakes, estuaries, rivers)^5^. Only islands where at least one monocot genus was present were included, regardless of the number of species found there. Although the study was designed at the genus level we were able to estimate sampling effort using a species richness approach (Supplementary Method S3). We used modeling to predict species richness and excluded islands where the observed species richness was 5 times lower than the predicted richness, which would be an indication of under-sampling. We chose this threshold because it allowed us to *i*) keep all monocot genera that occur on islands (we found that all genera from the islands that fell below the threshold were present on at least another island), *ii*) remove only islands with few genera (the maximum number of genera present on such islands was 8) and *iii*) avoid excluding complete clusters of spatially close islands. In the end, we were able to include 2,556,584 occurrences representing 15,964 species from 1,524 genera found on 4,306 islands. Crossing data from three large databases - the GBIF, eMonocot and UNEP-WCMC databases (2007), allowed us to compile, to our knowledge, the most comprehensive dataset comprising information on the largest number of islands used in island biogeography research.

### Metrics

We used the measure of phylogenetic endemism (PE) of Rosauer *et al*. (2009), with some adjustments to accommodate for certain features of our dataset, such as presence only data, and the aim of the study (Table 2). PE departs from the traditional definition of endemism as it does not represent the confinement of a species to a discrete geographic unit but measures the geographical concentration of evolutionary history compared to a broad landscape where diversity is distributed to varying degrees^17,19,20^. The PE index weights each branch of a phylogenetic tree by the inverse of the combined spatial range of all species it supports. Here, we replaced the spatial range by the number of islands on which a genus occurs. Thus, each branch length was weighted by the inverse of the number of islands harboring the genera supported by that branch. We then summed the weighted branch lengths joining those genera. This may be interpreted as PE on the scale of islands worldwide, which we called expanded insular phylogenetic endemism, PE_E_. At this stage, we did not take into consideration that a genus can occur both on islands and continents. Consequently, PE_E_ measures the PE of the set of genera present on each island.

**Table 2:** Metrics of phylogenetic endemism and associated categories.

Metric	Formula	Frequency at which the observed value is higher than the null model values				
		Significant endemism	Paleo-endemism	Neo-endemism	Mixed-endemism	Super-endemism
*PE*	$\sum _{c\in C}\frac{{l}_{c}}{{R}_{c}}$	≥0.95	≥0.95	≥0.95	≥0.95	≥0.99
		*OR*	*OR*	*OR*	*AND*	*AND*
*PEalt*	$\sum _{c\in Cnorm}\frac{l}{{R}_{c}}$	≥0.95	≥0.95	≥0.95	≥0.95	≥0.99
			*AND*	*AND*	*AND*	*AND*
*RPE*	$\frac{PE}{PEalt}$		≥0.95	≤0.05	>0.05 AND <0.95	>0.05 AND <0.95

This table shows the formulas of the metrics and the criteria for the establishment of each category of endemism. C is the set of branches in the path joining the taxa to the root of the tree, c is a branch in path C, and Lc is the length of branch c. Cnorm is C with all branches having the same length *l*. R_c_ as defined by Rosauer *et al*.^19^ is the sum of the range of all descendant taxa in a clade, here the number of islands on which a branch is present.

In a second step, we defined a measure of PE that gives more weight to genera that are only present on islands. We devised this measure to meet the objective of estimating the relative age of lineages having arisen on islands through diversification or relictualization but taking into account the specificities of the PE index. Branches supporting continental genera were weighted by the inverse of the maximum number of islands where a genus could be found (i.e. by the inverse of 4,306). We called this measure restricted phylogenetic endemism, PE_R_, which is approximately the PE of the set of genera only found on islands and absent from continents. With this weighting method, genera occurring on continents had very little influence on the value of insular phylogenetic endemism. This strategy avoids the exclusion of continental genera, which would have been misleading because *i*) some deep branches supporting genera that are island endemics also support genera occurring on continents, and *ii*) the differentiation between neo-, paleo-, and mixed endemism requires calculations based on the entire set of genera found on islands and not only on endemic ones (see the following section).

We used these two indices because they capture slightly different aspects of the distribution of phylogenetic endemism. The PE_E_ index, by considering genera occurring on continents, may help identify the abiotic conditions that may favour the establishment of either relatively ancient or recently evolved continental plants. The PE_R_ index on the other hand, may reflect speciation events on islands or past extinctions on the continent.

### Identifying areas of neo-, paleo-, mixed and super-endemism

To identify areas of neo-, paleo- and mixed endemism, relative phylogenetic endemism (RPE) was estimated for each island following the method of Mishler *et al*.^21^ (see also Table 2). For each island, we first calculated PE and an alternative measure of PE, called PEalt. To calculate PEalt, we modified the phylogeny by attributing a unique value to all branch lengths but kept the tree topology. We chose this value as the mean of all branch lengths of the original phylogeny (16.9 Ma)^53^. We then calculated RPE as the ratio between PE and PEalt (Table 2). To account for sampling bias^44,56^, the values of PE, PEalt and RPE were corrected using an index of geographic coverage (see Supplementary Method S3).

PE, PEalt and RPE values are dependent on species richness because the PE of a set of species increases when new species are added to the set. To circumvent this property, we used null models by randomizing genus occurrences among all islands 1,000 times (without replacement)^21,57^. This null model maintained the structure of the data: each island kept the same number of genera in each simulation and each genus kept the same number of occurrences across all islands. Following Mishler *et al*.^21^, we performed a two-tailed test to compare the observed value of all metrics with the simulated values for each island. An observed value that was higher than 95% of the simulated values was considered to be significantly high. Conversely, an observed value that was lower than 95% of the simulated values was considered to be significantly low (Table 2). A significant area of phylogenetic endemism is an island where its RPE numerator, denominator or both are significantly high (Table 2). These areas can be classified according to the following non-overlapping categories (Table 2)^21^:

- Areas of paleo-endemism are islands with a significantly high RPE ratio (i.e. PE is significantly higher than PEalt, meaning that long branches that are rare overall may be more common in that area)
- Areas of neo-endemism are islands with a significantly low RPE ratio (i.e. PE is significantly lower than PEalt, meaning that rare short branches may be more common)
- Areas of mixed endemism have a significantly high numerator and denominator, but the RPE is not significantly higher or lower than at random. These islands have a mix of rare long and rare short branches, and neither paleo-endemism nor neo-endemism is predominant.
- Super-endemism is a subdivision of this last category. It corresponds to islands where both the numerator and the denominator fall into the highest 1% of the distribution values obtained from simulations. Areas of super-endemism have a mix of either very rare short and long branches or of very long and very short rare branches).

This method was applied to PE_E_ and PE_R_. In the case of PE_R_, islands that did not harbor island-specific genera (they harbor only genera that occurr both on islands and continents) were excluded from the analysis. Thus, islands without endemic genera were not falsely identified as areas of restricted endemism. We carried out sensitivity tests for the identification of island age categories by gradually excluding islands in relation to their index of geographic coverage and by running analyses on these new sets of islands (described in Supplementary Method S3).

### Identifying the factors contributing to phylogenetic endemism

We selected 21 explanatory variables to explore the factors that may underly or be associated with the distribution of neo-, paleo-, mixed and super-endemism areas. Prior to carrying out our analyses, we calculated their pairwise correlation coefficients to avoid collinearity. Variables whose pairwise correlation coefficients were greater than 0.7 (Spearman test) were excluded from the analysis. These were temperature seasonality, isotherm, mean annual solar radiation, standard deviation in solar radiation, mean annual vapor pressure and standard deviation in wind speed.

The 15 variables included in the analysis were related to location, habitat availability, climatic and historical factors and as well as sampling effort (Table 3). A full description of the rationale for using these variables is provided in Supplementary Method S3.

**Table 3:** The 15 abiotic variables tested and data sources.

Variable	Unit	Source
**Localization**		
Latitude	Decimal degrees	UNEP-WCMC^55^
Longitude	Decimal degrees	UNEP-WCMC^55^
Minimum distance to continent	km	UNEP-WCMC^55^
Surrounding land mass proportion (SLMP)		Weigelt *et al*.^61^
**Habitat availability**		
Area	km²	UNEP-WCMC^55^
Elevation	m	Weigelt *et al*.^61^, UNEP-WCMC^55^
Number of ecoregions per island		Olson *et al*.^62^
**Climate**		
Mean annual temperature	°C	Fick & Hijmans^63^
Mean annual rainfall	mm	Fick & Hijmans^63^
Rainfall seasonality	mm	Fick & Hijmans^63^
Mean annual wind speed	m.s^−1^	Fick & Hijmans^63^
Standard deviation of vapor pressure	kPa	Fick & Hijmans^63^
**Historical factors**		
Connection to the mainland during the Last Glacial Maximum (GMMC)		Weigelt *et al*.^61^
Velocity of past climate change	y.m^−1^	Sandel *et al*.^64^
**Sampling effort**		
Relative Incidence Coverage Estimator (ICE_r_)		GBIF, Lee & Chao^65^

We used Boosted Regression Trees^58^ and multi-model selection^59^ to test for the effect of 15 variables on the occurrence of neo-, paleo-, mixed and super-endemism. These methods are complementary: BRT do not discriminate between response variables with low variation (e.g. Glacial Maximum Mainland Connection (GMMC)) whereas the multi-model selection algorithms do not handle missing values in the response variables, implying that many islands had to be excluded when it was performed (2,122 out of 4,306 islands were excluded). To calculate the relative contribution and influence of each variable on a category compared to the other variables, we attributed a value of 1 to the category being tested and a value of 0 to the others. We then assumed a binomial distribution of the data in both multi-model selection and Boosted Regression Trees. If the results from BRT and multi-model selection were not congruent, we used a generalized linear model to test conflicting variables in turn. This helped control some sources of uncertainty in the results of BRT or multi-model selection (such as the effects of possible remaining collinearity, despite having removed variables with r > 0.7) and helped determine which results were most likely. All analyses were performed with R version 3.4.0 and packages gbm^60^ and MuMin^59^. A full description of the methods can be found in Supplementary Method S3.

## Supplementary information

Supplementary Dataset 1

Supplementary Dataset 2

Supplementary Dataset 3

Supplementary material

## Data Availability

All insular monocot occurrences are available in Dryal Deposital Repository (data will be made available following acceptance). Other data (phylogeny, island database) are accessible from the references or upon request to their authors.
