# Supplementary material for: Distribution and relative age of endemism across islands worldwide
Source: Sci Rep. 2019 Aug 12;9:11693. doi: 10.1038/s41598-019-47951-6 (PMC6690940; doi:10.1038/s41598-019-47951-6)
Supplement: Supplementary file 4 — Supplementary material [file 41598_2019_47951_MOESM4_ESM.pdf]

Distribution and relative age of endemism across islands worldwide

Simon Veron[1,2*], Thomas Haevermans[1], Rafaël Govaerts[3], Maud Mouchet[2] and Roseli Pellens[1]

[1] *Institut de Systématique, Evolution, Biodiversité (ISYEB), Muséum national d'Histoire naturelle, CNRS, Sorbonne Université, EPHE, CP 51, 47 rue Buffon, 75005 Paris, France*
[2]*Centre d'Ecologie et des Sciences de la Conservation (CESCO UMR7204) MNHN, CNRS, Sorbonne Université - CP51, 55-61 rue Buffon, 75005, Paris, France*
[3]*Royal Botanic Gardens, Kew, Richmond, Surrey TW9 3AE, UK.*

Supplementary Figure S1: fitted functions estimated from Boosted Regression Trees

(a) Expanded PE

1. Significant areas of endemism

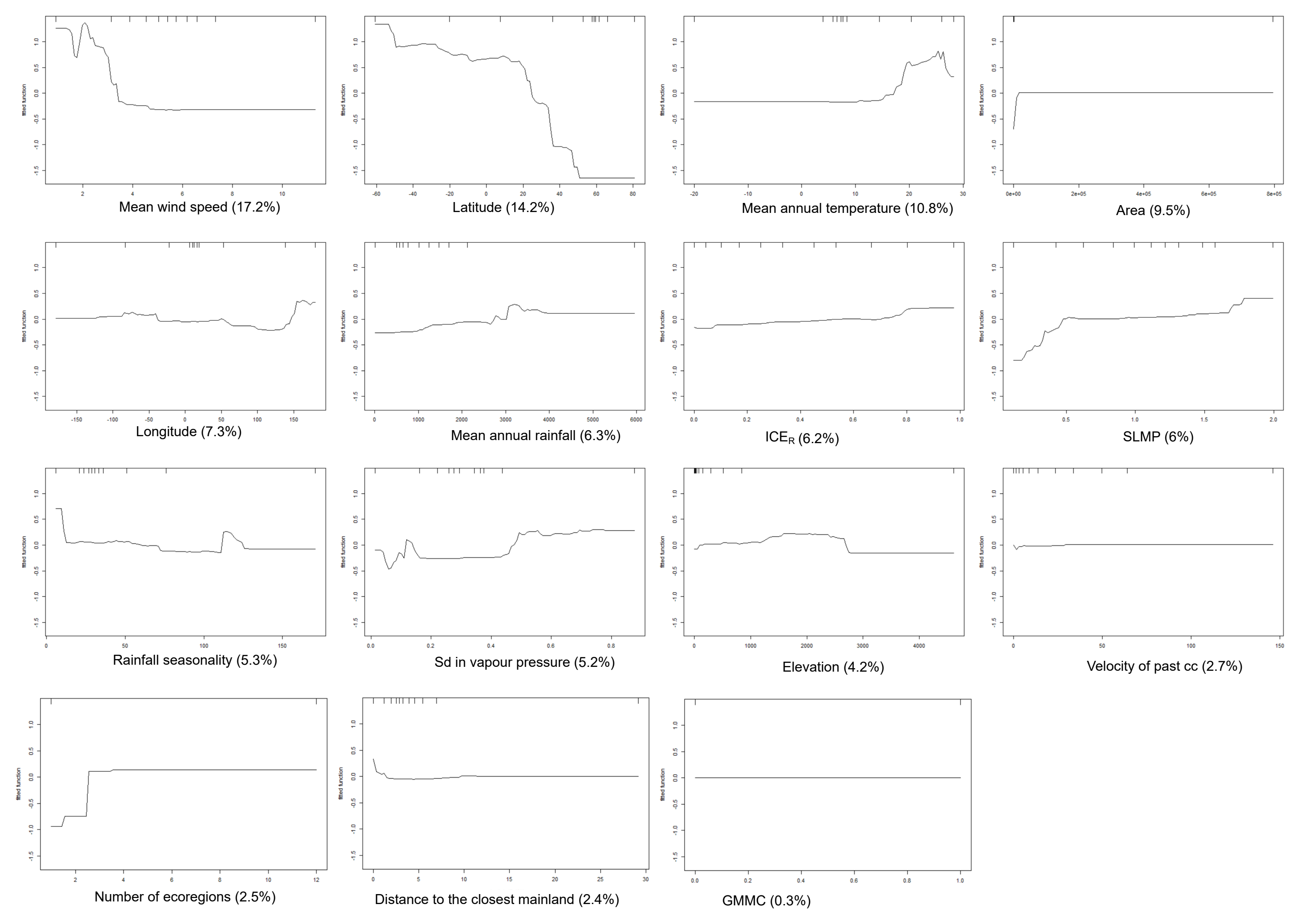

2. Paleo-endemism

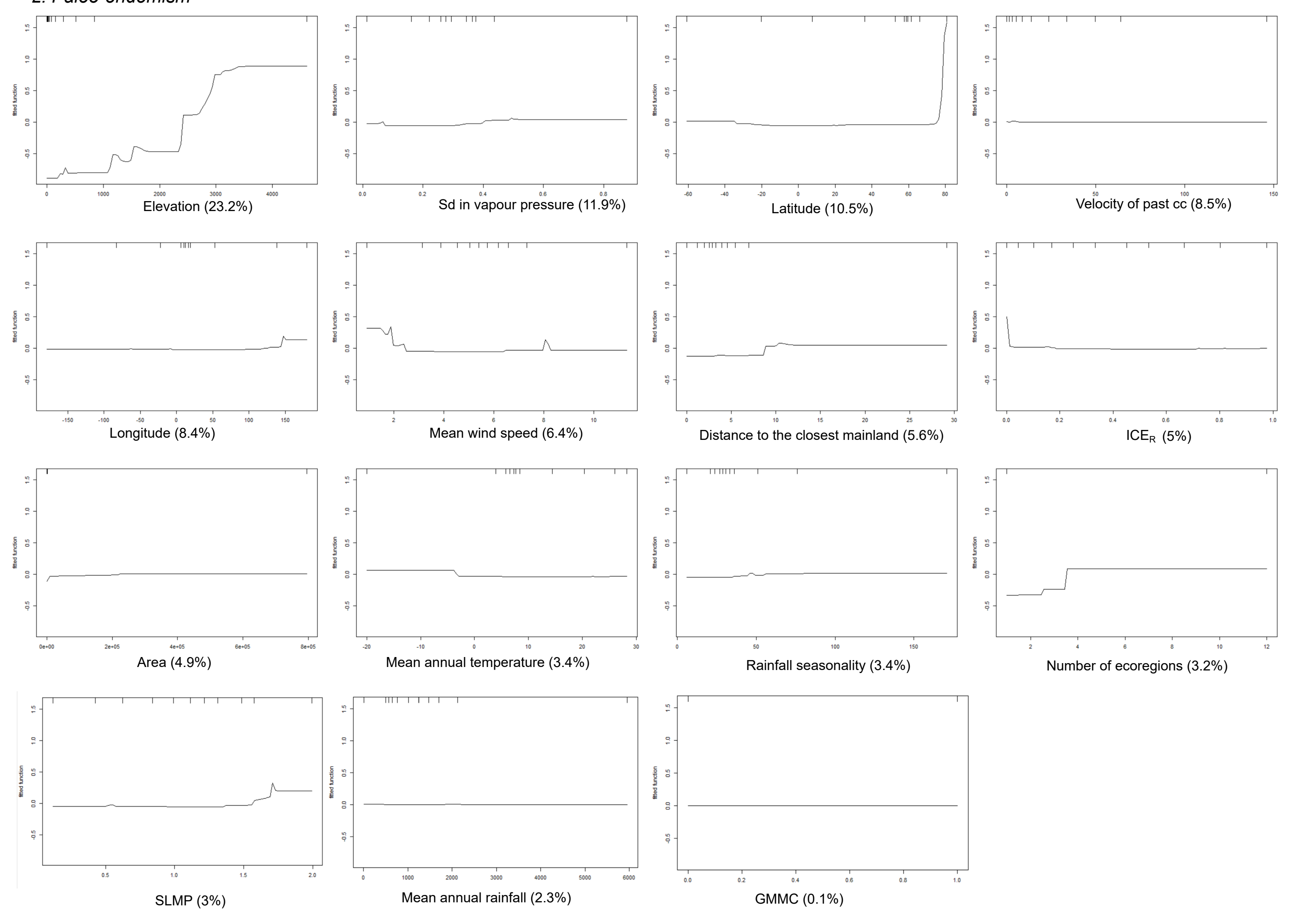

3. Mixed-endemism

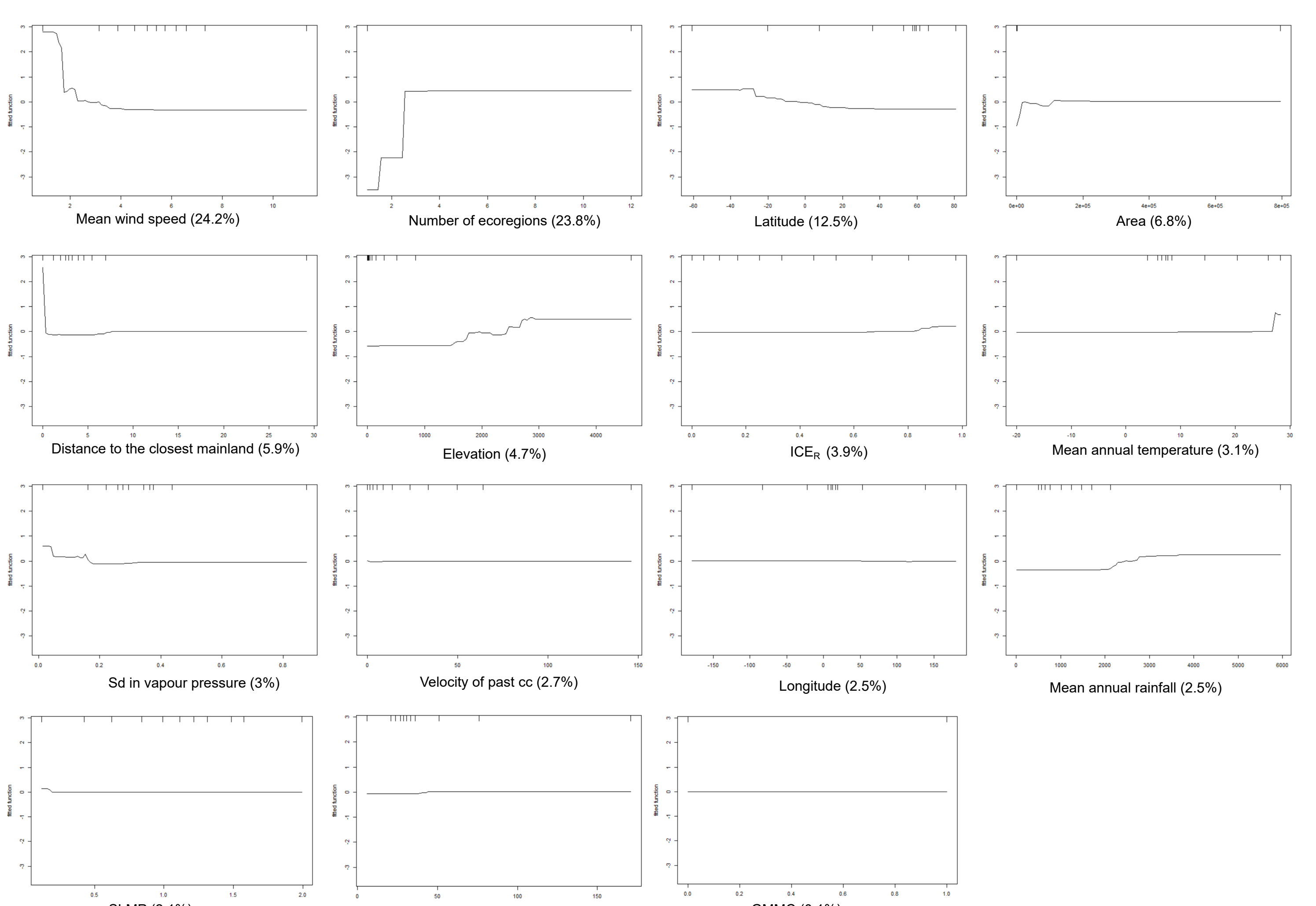

4. Super-endemism

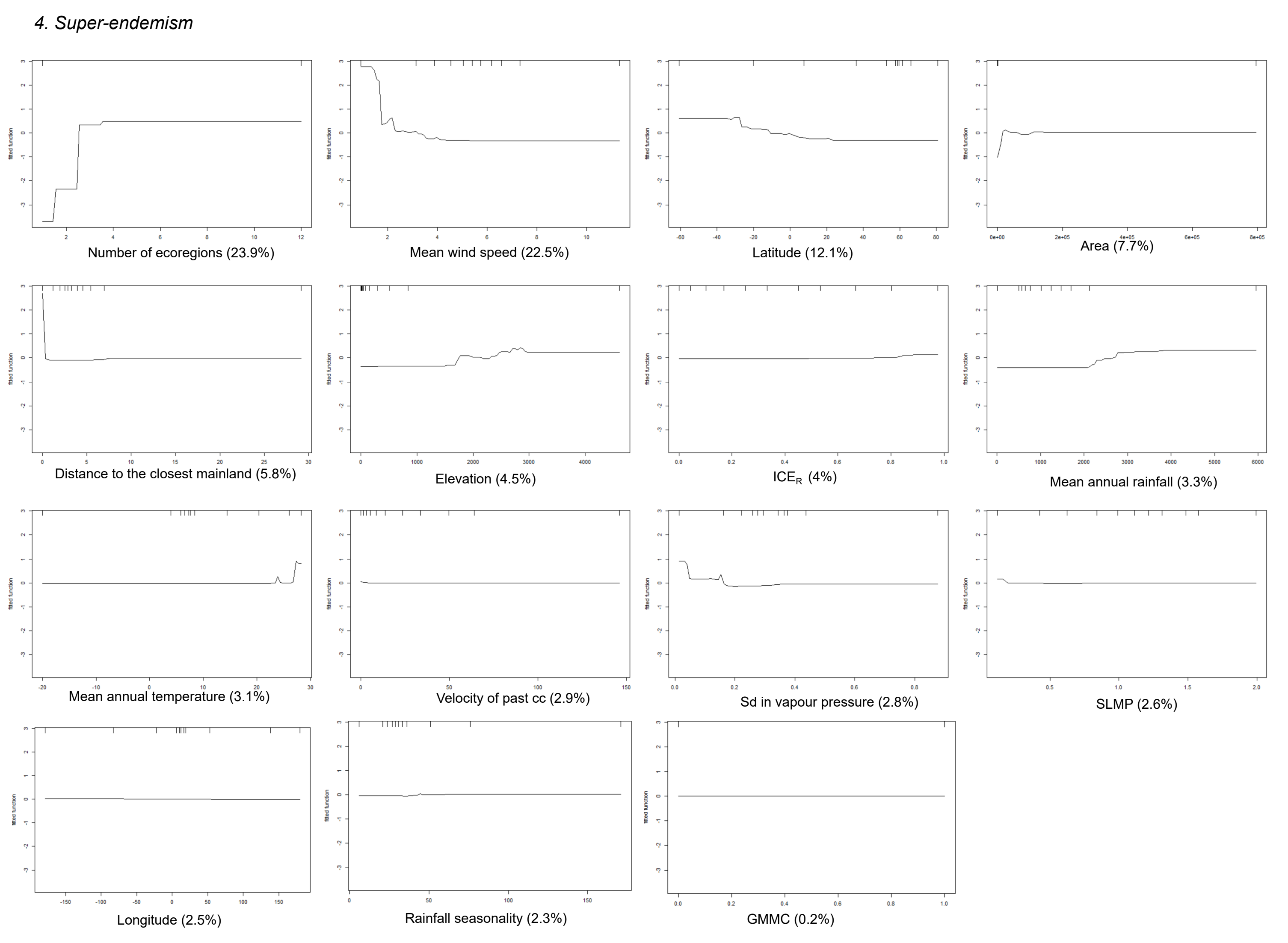

(b) Restricted PE

1. Significant areas of endemism

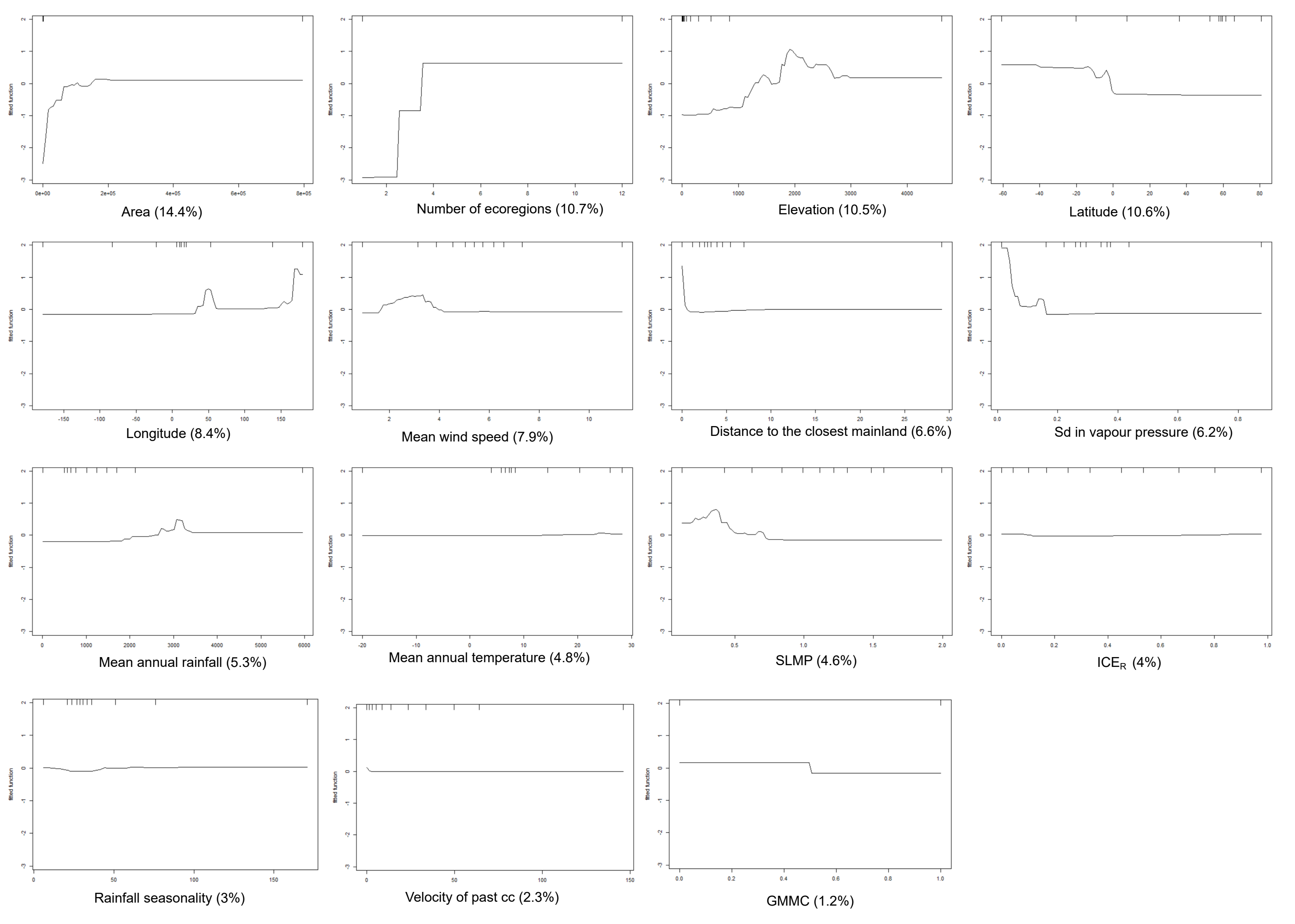

2. Paleo-endemism

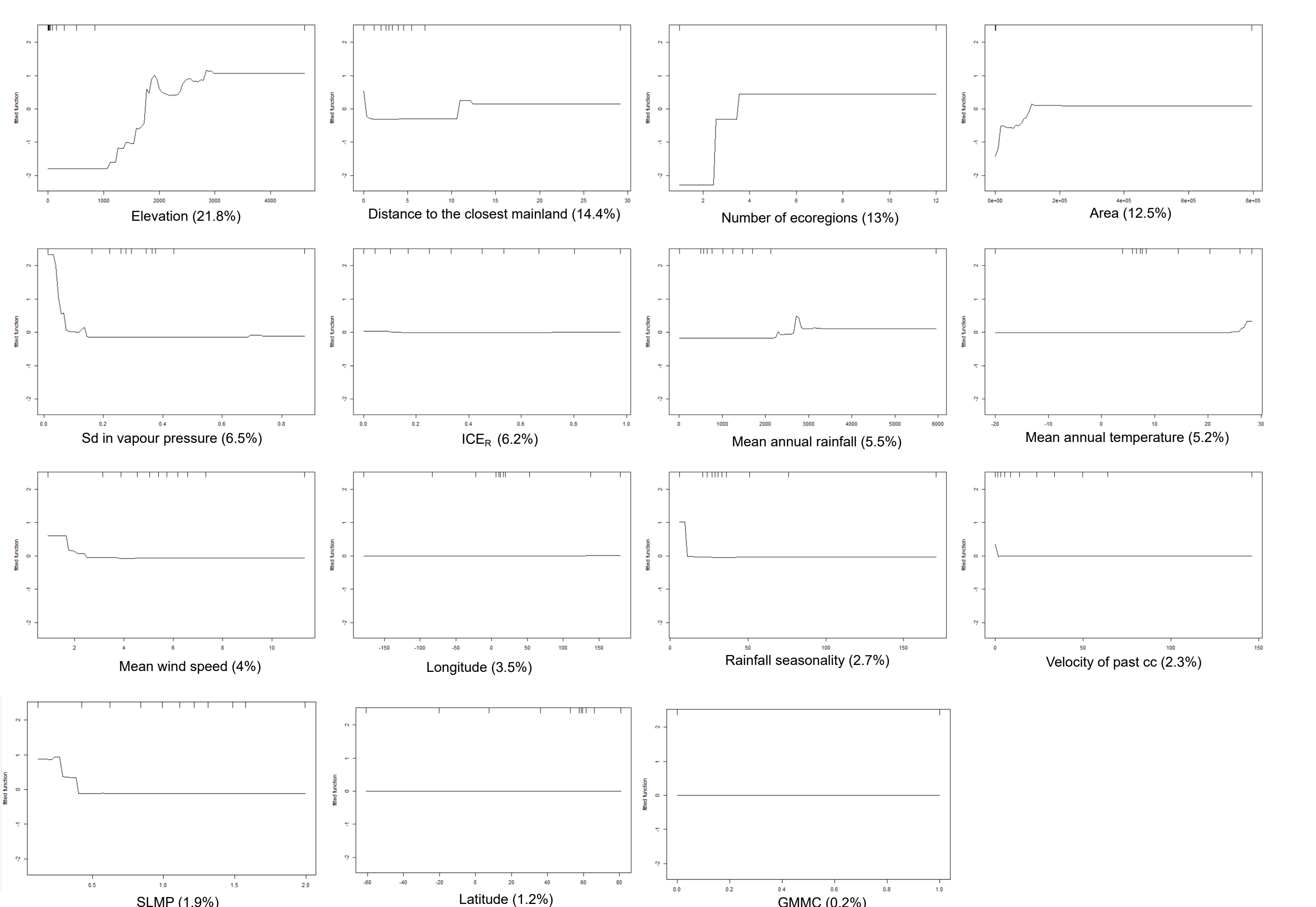

3. Mixed-endemism

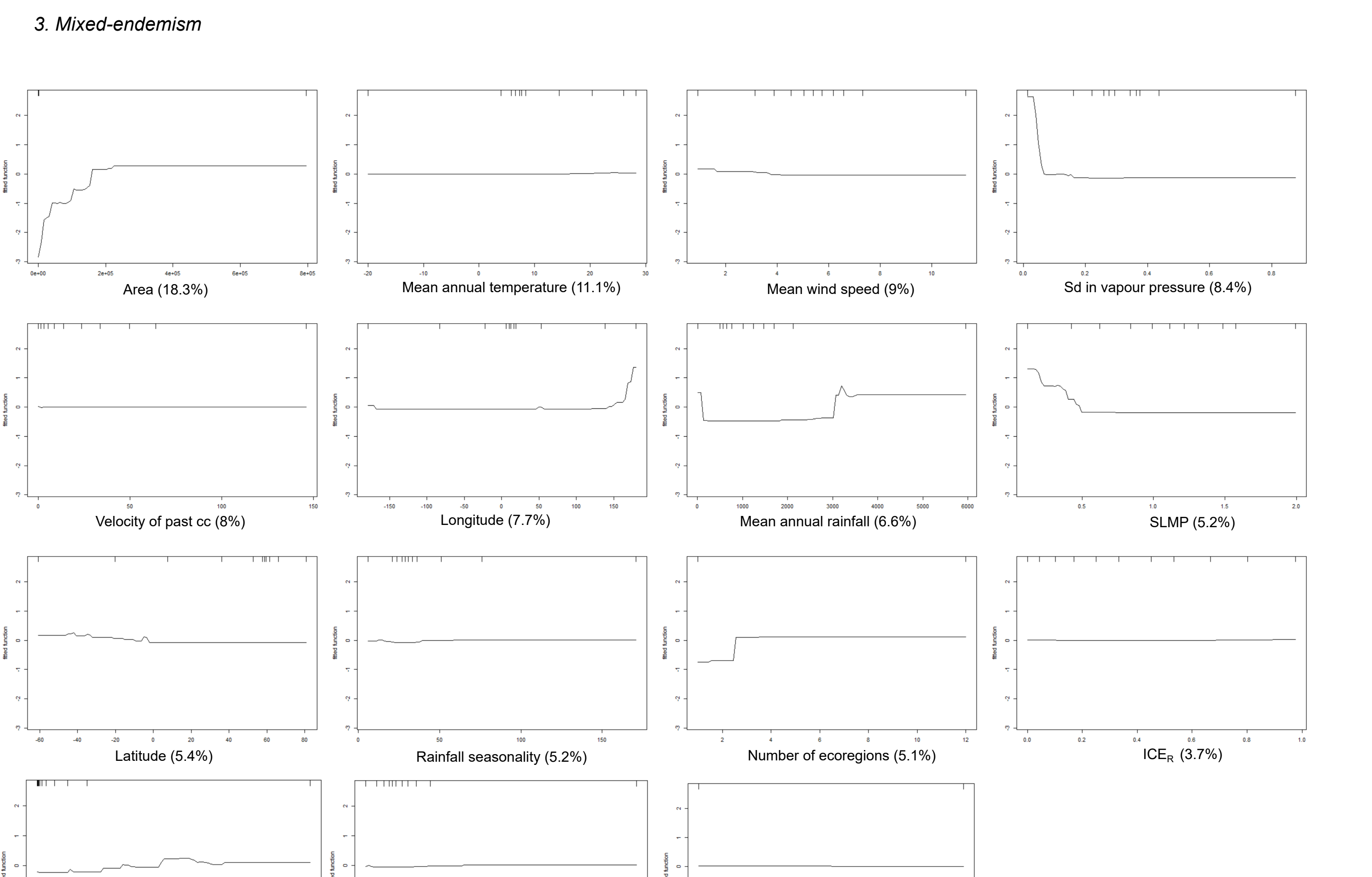

4. Super-endemism

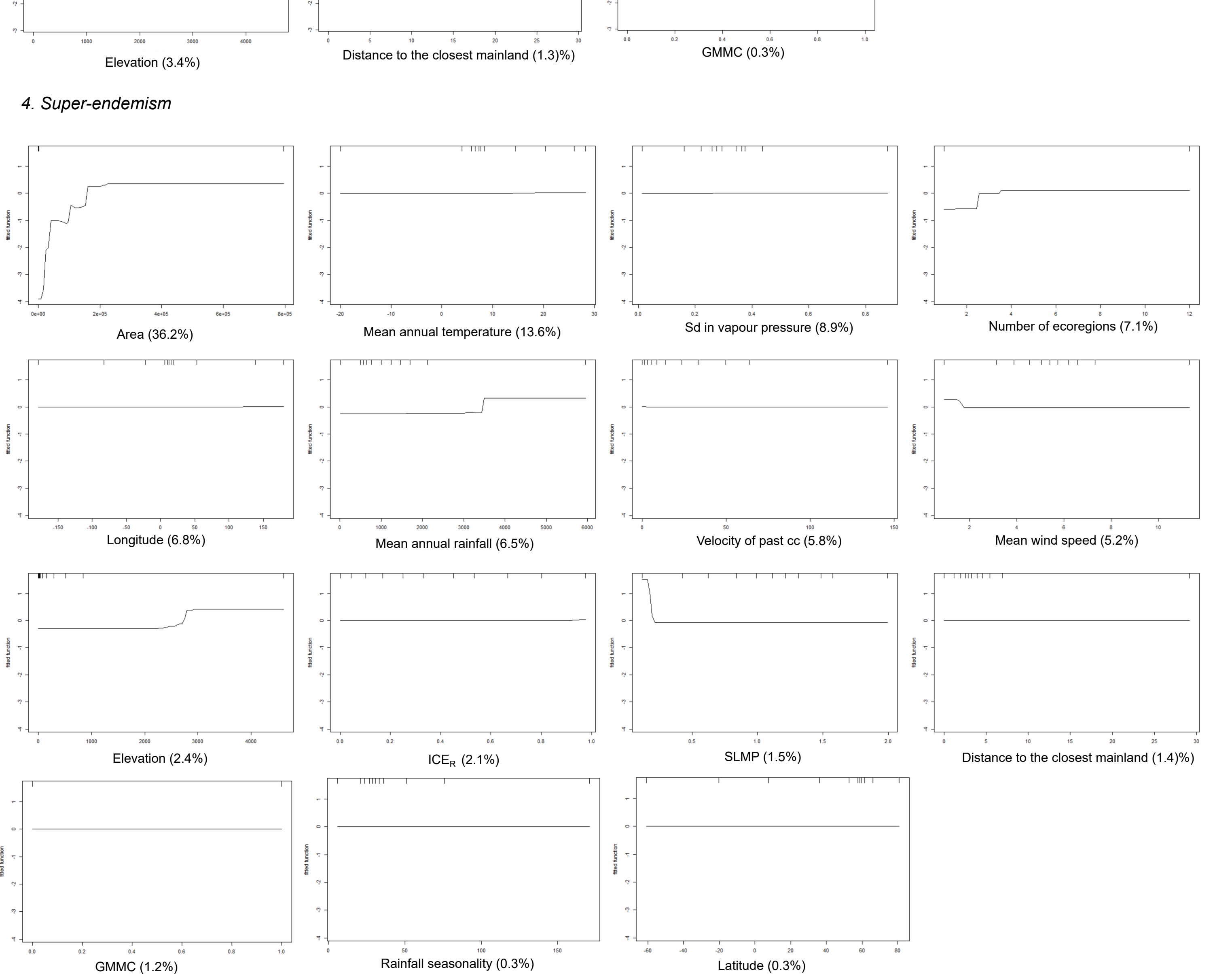

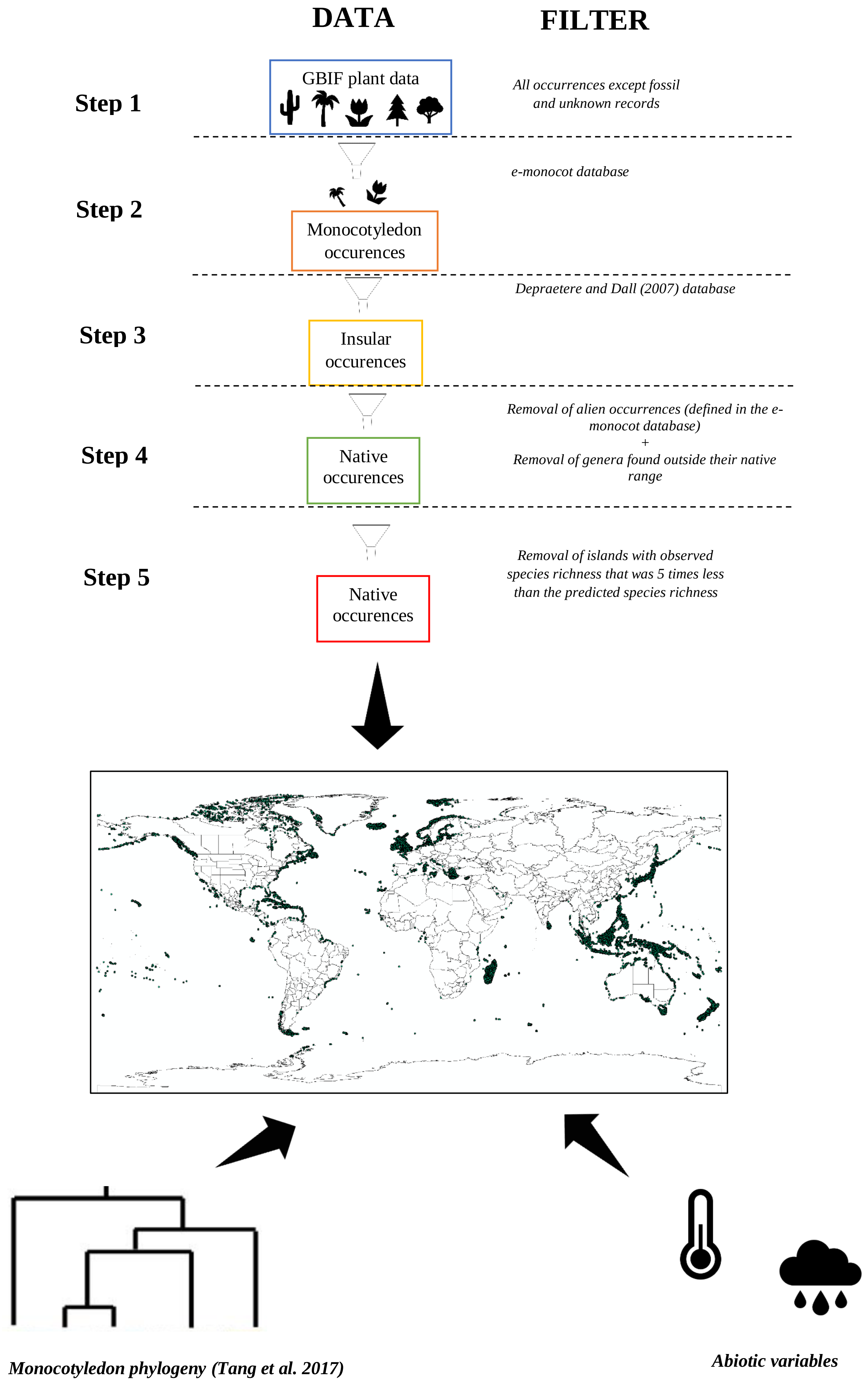

Supplementary Figure S2: Selection procedure of GBIF data.

Supplementary Method S3: Supplementary materials and methods

A. Corrected PE, PEalt and RPE values - addendum

A limitation in the GBIF data is the difference in geographic coverage between islands[1]. We took this uncertainty into account using a two-step procedure. First, we calculated an index of geographic coverage for each island. To do so, we used modelling to predict species richness on each island. We estimated how species number on an island varied according to a combination of bioclimatic, geographic and historical variables using Boosted Regression Trees (see Supplementary Method S3.B for a full description of these variables). Boosted Regression Trees is an ensemble method for fitting statistical models which combines regression trees and boosting algorithms [2]. The advantages of Boosted Regression Trees are described in Supplementary Method S3.D. We chose an optimal number of trees based on learning rate, tree complexity and bag fraction (generally lr = 0.001; tree.complexity = 5; bg = 0.75) to predict values of species richness. With this model we predicted species richness for each island using the *predict* [3]. The index of geographic coverage for each island was obtained as the ratio of the observed value of species richness over the predicted values.

Secondly, we performed linear models (negative binomial distribution) of PE and PEalt as a function of our index of geographic coverage. Corrected values of PE and PEalt were defined as the residuals of this linear model and corrected RPE values were calculated as their ratio. We performed calculations to correct both PE_E and PE_R. However, the identification of areas of neo-, paleo-, mixed and super-endemism already relied on null models that removed dependency on species richness. We therefore did not correct, with our index of geographic coverage, the calculations that determined which category of endemism an area belongs to. Doing so would have included circularity when looking for predictors of phylogenetic endemism.

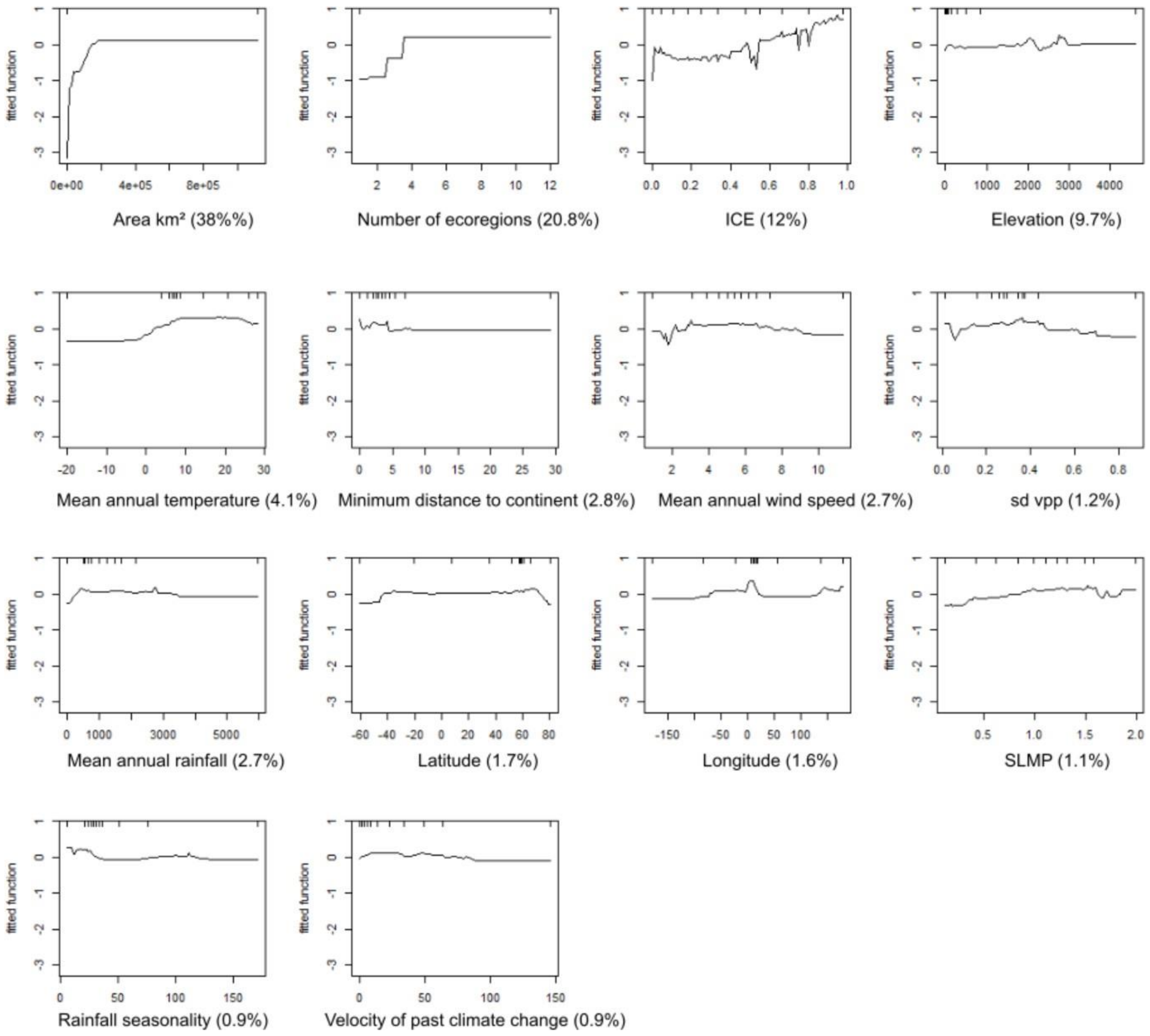

Fig. Contribution of abiotic variables to species richness used for predictions of geographic coverage. Vpp= vapour pressure; ICEr=Incidence Coverage Index; SLMP= Proportion of Surrounding Land Mass; sd=standard deviation

B. Details of the selected abiotic variables

To explore which factors could explain the distribution of areas of neo-, paleo-, mixed and super-endemism we tested variables related to:

i. Localization: minimum distance from the continent (km), surrounding landmass proportion (SLMP), latitude, and longitude.

   Species diversity theoretically increases with proximity to the continent, mainly because of colonization [4]. The distance from the mainland is one of the strongest

predictors of plant species richness on islands [5]. However, despite having low species richness, remote islands may harbor high rates of endemism due to in situ speciation [6]. SLMP, which accounts for the size and shape of the coastline of the surrounding landmass, has been found to be a valuable isolation metric to explain island plant diversity on a global scale [7]. Latitude and longitude may highlight differences related to geographic position, especially along the Latitudinal Diversity Gradient [8].

Latitude, longitude, and minimum distance from the continent were obtained from the UNEP-WCMC database [9]. SLMP came from Weigelt et al. [10].

ii. Habitat availability: area (km^2), elevation (meters), and number of ecoregions on an island. Species richness and speciation rates theoretically increase with area [4], which is among the strongest predictors of plant species richness [5]. Maximal elevation is related to topography and environmental heterogeneity as, for instance, evident from the temperature decrease with altitude [10] [11]. Elevation has also been shown to account to a large extent for insular plant species richness [5]. The number of ecoregions per island is defined as the number of areas with distinct environmental conditions and distinct assemblages of natural communities sharing a large majority of species [12].

Island area was obtained from the UNEP-WCMC database [9]. Elevation was extracted from Weigelt et al. [10] and the number of ecoregions per island was obtained from Olson et al. [12].

iii. Bioclimatic factors: mean annual temperature (C°), mean annual rainfall (mm), temperature seasonality (C°), rainfall seasonality (mm), mean annual solar radiation (kJ m^{-2} day^{-1}) and its standard deviation (sd), mean annual wind speed (m s^{-1}) and its sd, mean annual water vapor pressure (kPa) and its sd, and isotherm

(C°). Bioclimatic factors may select against lineages adapted to certain environmental conditions, a process called environmental filtering [13]. Temperature and rainfall were shown to be among the main predictors of plant species richness [5 14 15]. Wind speed may act on plant diversity by favoring long-distance dispersal [16], influencing plant growth [17], and selecting for lineages adapted to cool or harsh wind conditions. Evapotranspiration, measured from water vapor pressure, and solar radiation are key components related to energy availability. They were both shown to be predictors of plant richness on islands as well as on continents [14 15 16]. Temperature, rainfall, wind speed, solar radiation and vapor pressure data were extracted from Worlclim version 2 [19], and missing data were completed with the GID [9] database.

iv. Historical factors: connection to continental areas during the Last Glacial Maximum (Glacial Maximum Mainland Connection GMMC) and velocity of past climate change. GMMC discriminates roughly between continental and oceanic islands. Oceanic islands are lifeless at birth, then colonized. By contrast, continental islands are fragments of continental areas where life was already present. The effect of past climate change velocity may depend on the taxon considered, however low velocity is generally associated with high rates of endemism on a regional scale [20]. Data on island age, area and isolation during the Last Glacial Maximum were not available for a sufficient number of islands to be included in the analysis. GMMC data were extracted from Weigelt et al. [10] and velocity of past climate change from Sandel et al. [20].

v. Sampling effort: the ICE*r* metric described in Supplementary Method S3.C.

By testing collinearity between variables we excluded from our analysis variables with a Spearman coefficient higher than 0.5, i.e. temperature seasonality, isotherm, mean annual

solar radiation, standard deviation in solar radiation, mean annual vapor pressure and standard deviation in wind speed.

C. Estimating sampling effort

To estimate sampling effort we chose the Incidence-based Coverage Estimator (ICE [21]) which is calculated from the number of rare species in a sample and from species accumulation curves. Compared with other estimators of species richness, ICE may best satisfy the requirements of an ideal species-richness estimator, including the capacity to reach a stable value independently of sample size, and low sensitivity to sampling patchiness and density. We calculated ICE by defining a sub-sample within an island as a set of observations obtained at a given date, and using the R function spp.est from the 'Fossil' package [22]. We then calculated the ICE_r index as the ratio of the observed number of species in an area over the expected number of species estimated with ICE.

D. Correlation between variables

	**Area**	**Longitude**	**Latitude**	**velocity**	**Temp_mean**	**Rainf_mean**
Area	1	0,0582958	-0,05166	-0,038944	0,023055997	0,098353712
Longitude	0,058295757	1	-0,480516	-0,132796	0,371554542	0,157280381
Latitude	-0,051659852	-0,480516	1	0,4295239	-0,693353609	-0,23549543
velocity	-0,038943812	-0,132796	0,4295239	1	-0,393970367	-0,31065206
Temp_mean	0,023055997	0,3715545	-0,693354	-0,39397	1	0,38406983
Rainf_mean	0,098353712	0,1572804	-0,235495	-0,310652	0,38406983	1
ICEr	0,092333471	-0,030764	0,1017696	0,0147821	-0,140160315	-0,09524605
min.dist	-0,004851742	-0,169117	0,1717789	-0,030273	-0,339646497	-0,06296626
Temp_seas	-0,016812743	-0,298291	0,641296	0,4881331	**-0,788587614**	-0,51580821
Rainf_seas	-0,00606701	0,089763	-0,302624	-0,312024	0,473021098	-0,13088943
Isotherm_mean	0,088732486	0,2459469	**-0,707355**	-0,454954	**0,816841837**	0,476516901
wind_mean	-0,117055486	-0,137321	0,3666328	0,2686721	-0,558062368	-0,25361898
wind_sd	-0,064708584	-0,032507	0,2932016	0,0822503	-0,314860326	-0,0002615
srad_mean	0,046074405	0,4875739	**-0,965793**	-0,453703	**0,759610828**	0,218050725
srad_sd	-0,085989639	-0,222518	0,584389	0,4361127	**-0,821556196**	-0,5302112
vpp_mean	0,061471046	0,4126318	**-0,767464**	-0,418117	**0,902416909**	0,420346816
vpp_sd	-0,082358126	0,0175722	0,187376	0,273649	-0,007864785	-0,29195887
SLMP	-0,108792257	-0,237639	0,5795588	0,5086212	-0,463119935	-0,34747215
GMMC	-0,002714113	-0,06863	0,2988371	0,3506478	-0,257257985	-0,13435347
Elev	0,449757896	0,0320983	-0,143862	-0,304957	0,104344339	0,235445783
Nb_ecoreg	0,47687125	-0,038353	-0,086733	-0,0977	0,12873637	0,093178158

	ICEr	min.dist	Temp_seas	Rainf_seas	Isotherm_mean	
Area	0,09233347	-0,004852	-0,016813	-0,006067	0,08873249	
Longitude	-0,03076374	-0,169117	-0,298291	0,089763	0,24594691	
Latitude	0,10176963	0,1717789	0,641296	-0,302624	**-0,70735515**	
velocity	0,0147821	-0,030273	0,4881331	-0,312024	-0,45495441	
Temp_mean	-0,14016031	-0,339646	**-0,788588**	0,4730211	**0,81684184**	
Rainf_mean	-0,09524605	-0,062966	-0,515808	-0,130889	0,4765169	
ICEr	1	-0,002722	0,0803965	-0,054367	-0,06977914	
min.dist	-0,00272153	1	0,206397	0,0187914	-0,21373255	
Temp_seas	0,08039648	0,206397	1	-0,214541	**-0,82001456**	
Rainf_seas	-0,05436739	0,0187914	-0,214541	1	0,33041639	
Isotherm_mean	-0,06977914	-0,213733	**-0,820015**	0,3304164	1	
wind_mean	0,10560573	0,1982152	0,2552347	-0,396698	-0,57708381	
wind_sd	0,08725226	0,2108832	0,0117421	-0,194866	-0,30156485	
srad_mean	-0,10538601	-0,145233	-0,647047	0,4168445	**0,75125801**	
srad_sd	0,11884912	0,1188521	**0,7192312**	-0,383476	**-0,85216128**	
vpp_mean	-0,13823354	-0,155206	-0,766299	0,47109	**0,88034716**	
vpp_sd	-0,05300385	-0,114263	0,4470566	0,1976905	-0,42893387	
SLMP	0,03984488	0,0010492	0,5846143	-0,112137	**-0,57257336**	
GMMC	0,01676547	0,0395843	0,3034807	-0,065749	-0,36579737	
Elev	0,12436883	-0,045156	-0,185144	0,0314747	0,25156285	
Nb_ecoreg	0,10217834	-0,088918	-0,122901	0,0103442	0,19657519	
	**wind_mean**	**wind_sd**	**srad_mean**	**srad_sd**	**vpp_mean**	
Area	-0,117055486	-0,064709	0,0460744	-0,08599	0,06147105	
Longitude	-0,137321363	-0,032507	0,4875739	-0,222518	0,41263182	
Latitude	0,366632814	0,2932016	**-0,965793**	0,584389	**-0,76746414**	
velocity	0,268672059	0,0822503	-0,453703	0,4361127	-0,41811691	
Temp_mean	-0,558062368	-0,31486	**0,7596108**	**-0,821556**	**0,90241691**	
Rainf_mean	-0,253618982	-0,000262	0,2180507	-0,530211	0,42034682	
ICEr	0,105605734	0,0872523	-0,105386	0,1188491	-0,13823354	
min.dist	0,198215197	0,2108832	-0,145233	0,1188521	-0,15520646	
Temp_seas	0,255234708	0,0117421	-0,647047	**0,7192312**	**-0,76629891**	
Rainf_seas	-0,396697698	-0,194866	0,4168445	-0,383476	0,47109001	
Isotherm_mean	-0,57708381	-0,301565	**0,751258**	**-0,852161**	**0,88034716**	
wind_mean	1	**0,7408038**	-0,439845	0,5705991	-0,59486051	
wind_sd	**0,740803819**	1	-0,303578	0,2420419	-0,3086409	
srad_mean	-0,439844608	-0,303578	1	-0,64128	**0,83338527**	
srad_sd	0,570599136	0,2420419	-0,64128	1	**-0,8708028**	
vpp_mean	-0,594860515	-0,308641	**0,8333853**	**-0,870803**	1	
vpp_sd	-0,006043055	-0,122555	-0,140731	0,1493892	-0,19910154	
SLMP	0,09743942	-0,049383	-0,579355	0,5634297	-0,52804495	
GMMC	0,155691382	0,0899011	-0,302205	0,2985624	-0,28895638	
Elev	-0,162574692	-0,022815	0,1258057	-0,235728	0,09648475	
Nb_ecoreg	-0,160979611	-0,102393	0,1014168	-0,174516	0,11845969	
	**vpp_sd**	**SLMP**	**GMMC**	**Elev**	**Nb_ecoreg**	
Area	-0,082358126	-0,108792	-0,002714	0,4497579	0,47687125	
Longitude	0,017572197	-0,237639	-0,06863	0,0320983	-0,03835331	
Latitude	0,187375963	0,5795588	0,298837	-0,143862	-0,08673327	

velocity	0,273648966	0,5086212	0,3506478	-0,304957	-0,09769956	
Temp_mean	-0,007864785	-0,46312	-0,257258	0,1043443	0,12873637	
Rainf_mean	-0,291958866	-0,347472	-0,134353	0,2354458	0,09317816	
ICEr	-0,053003852	0,0398449	0,0167655	0,1243688	0,10217834	
min.dist	-0,114262595	0,0010492	0,0395843	-0,045156	-0,0889181	
Temp_seas	0,447056605	0,5846143	0,3034807	-0,185144	-0,12290089	
Rainf_seas	0,197690485	-0,112137	-0,065749	0,0314747	0,01034424	
Isotherm_mean	-0,428933867	-0,572573	-0,365797	0,2515629	0,19657519	
wind_mean	-0,006043055	0,0974394	0,1556914	-0,162575	-0,16097961	
wind_sd	-0,122555244	-0,049383	0,0899011	-0,022815	-0,10239348	
srad_mean	-0,140731405	-0,579355	-0,302205	0,1258057	0,10141684	
srad_sd	0,149389171	0,5634297	0,2985624	-0,235728	-0,17451586	
vpp_mean	-0,199101537	-0,528045	-0,288956	0,0964848	0,11845969	
vpp_sd	1	0,3286121	0,2635913	-0,259226	-0,09803034	
SLMP	0,328612112	1	0,6384111	-0,368979	-0,16252893	
GMMC	0,263591334	0,6384111	1	-0,303651	-0,10541214	
Elev	-0,259226282	-0,368979	-0,303651	1	0,38474403	
Nb_ecoreg	-0,098030339	-0,162529	-0,105412	0,384744	1	

Table. Pearson's correlation coefficient between the 21 variables considered (before that 6 of them were discarded due to absolute correlation>0.7). Temp=Temperature; Rainf=rainfall; srad=solar radiation; vpp=vapour pressure; min.dist=minimum distance to the continent; Elev=Elevation; seas=seasonality; sd=standard deviation

E. Modelling the contribution and significance of abiotic variables on categories of endemism

We use both Boosted Regression Trees and multi-model selection to test for the effect of abiotic variables on the identification of areas of neo-, paleo-, mixed and super-endemism.

Each endemism category was first tested independently using Boosted Regression Trees and assuming binomial distributions: the tested category was attributed a value of 1 whereas other categories were attributed a value of 0. Boosted Regression Trees is an ensemble method for fitting statistical models which combines regression trees and boosting algorithms [2]. The advantages of Boosted Regression Trees include: robust parameter estimations as a result of the integrated stochastic gradient boosting algorithm; model structure is learned from the data and not determined a priori, thereby avoiding assumptions required for model specification;

and easy implementation of complex and/or multi-way interactions. We first looked for the best combination of learning rate, tree complexity and bag fraction in order to select for the minimum number of trees that achieved minimum prediction error [2]. Learning rate is used to shrink the contribution of each tree as it is added to the model, tree complexity is the number of nodes in a tree and the bag fraction is the proportion of randomly selected data at each step for model fitting. Ideally, low learning rates and a tree complexity that reflects the true interaction order of the response being modelled (which is almost always unknown) should be used [2]. The bag fraction should be within the 0.5 - 0.75 range. To allow for tree convergence a minimum of 1,000 trees is recommended by Elith et al. [2]. To satisfy these criteria we generally chose a learning rate of 0.005, a tree complexity of 5 and a bag fraction of 0.5. Boosted Regression Trees algorithms were run with the 'dismo' package in [23].

To test for the significance and the direction of the relationship between category of endemism and our set of variables, we used generalized linear models assuming a binomial data distribution. We ran one model for each possible combination of the tested variables using the dredge function of the MuMIn R package version 1.9.13 [24]. However, we had to remove missing values to run multi-model selection and thus tested these models on a subset of 2,184 islands. The most complex model included the fixed additive effects of localization, habitat availability, bioclimatic, historical and sampling efforts. We then generated a set of best models, selected on the basis of Akaike's Information Criteria (AIC; adjusted for small sample size, i.e. AICc). The lower the AICc, the better the model. The 'best model set' was defined as the model set for which cumulative AICc weight (*w*, i.e. a measure of relative statistical support) reached 95% of the total AICc weights. Parameter estimates were averaged across the selected models using the model averaging function (full average [24]). This procedure enabled us to account for model selection uncertainty. All statistical analyses were performed under R version 3.4.0 [3].

F. Sensitivity analysis based on variation in geographic coverage

Specifically, we ran our analysis on data-sets where islands with an index of predicted species richness inferior to 0.25, 0.50 and 0.75 were excluded. With these data-sets, nearly all islands identified previously as priority areas of endemism were again selected as such, i.e. in these 3 data-sets only 5,6 and 7 priority islands were not selected. Moreover, changes in category of endemism was minor (Table A1). The table shows that changes are more frequent in the "super-endemism" category. This was expected because the p-value required to be classified in the "super" category is very high: as our method is relative, even little change in the p-value caused by changes in species occurrence data may influence the selection of "super-endemism" areas. However, among these islands not selected as "Super" in the sensitivity analysis, a high proportion of them were actually identified as "Mixed" which is in accordance with the fact that the "super" category is part of the "mixed" category.

	**Index of coverage of excluded islands**		
	**<0.25**	**<0.50**	**<0.75**
**Mixed**	0.17	0.26	0.2
**Paleo**	0	0.07	0
**Neo**	0.4	0.33	0.2
**Super**	0.36	0.36	0.36

Table: proportion of category change when islands were excluded depending on an index of coverage

References cited in this Supplementary Method

1. Meyer, C., Weigelt, P. & Kreft, H. Multidimensional biases, gaps and uncertainties in global plant occurrence information. *Ecology Letters* **19**, 992–1006 (2016).
2. Elith, J., Leathwick, J. R. & Hastie, T. A working guide to boosted regression trees. *Journal of Animal Ecology* **77**, 802–813 (2008).
3. R Core Team (2019). R: A language and environment for statistical computing. R Foundation for Statistical Computing, Vienna, Austria. http://www.R-project.org/
4. MacArthur, R. H. & Wilson, E. O. *The theory of island biogeography*. (Princeton, NJ: Princeton UniversityPress, 1967).

5. Kreft, H., Jetz, W., Mutke, J., Kier, G. & Barthlott, W. Global diversity of island floras from a macroecological perspective. *Ecology Letters* **11**, 116–127 (2008).
6. Gillespie, R. G. Oceanic islands: models of diversity. *Encyclopedia of biodiversity* 1–13 (2007).
7. Weigelt, P. & Kreft, H. Quantifying island isolation - insights from global patterns of insular plant species richness. *Ecography* **36**, 417–429 (2013).
8. Pianka, E. R. Latitudinal Gradients in Species Diversity: A Review of Concepts. *The American Naturalist* **100**, 33–46 (1966).
9. UNEP-WCMC. Global islands database. United Nation's Environmental Program, Cambridge, United Kingdom. (2013).
10. Weigelt, P., Jetz, W. & Kreft, H. Bioclimatic and physical characterization of the world's islands. *Proceedings of the National Academy of Sciences* **110**, 15307–15312 (2013).
11. Allouche, O., Kalyuzhny, M., Moreno-Rueda, G., Pizarro, M. & Kadmon, R. Area-heterogeneity tradeoff and the diversity of ecological communities. *Proceedings of the National Academy of Sciences* **109**, 17495–17500 (2012).
12. Olson, D. M. *et al.* Terrestrial Ecoregions of the World: A New Map of Life on Earth. *BioScience* **51**, 933 (2001).
13. Webb, C. O., Ackerly, D. D., McPeek, M. A. & Donoghue, M. J. Phylogenies and Community Ecology. *Annual Review of Ecology and Systematics* **33**, 475–505 (2002).
14. Currie, D. J. Energy and Large-Scale Patterns of Animal- and Plant-Species Richness. *The American Naturalist* **137**, 27–49 (1991).
15. Kreft, H. & Jetz, W. Global patterns and determinants of vascular plant diversity. *Proceedings of the National Academy of Sciences* **104**, 5925–5930 (2007).

16. Soons, M. B. & Ozinga, W. A. How important is long-distance seed dispersal for the regional survival of plant species?: Importance of long-distance seed dispersal. *Diversity and Distributions* **11**, 165–172 (2005).

17. Drake, B. G., Leadley, P. W., Arp, W. J., Nassiry, D. & Curtis, P. S. An Open Top Chamber for Field Studies of Elevated Atmospheric CO 2 Concentration on Saltmarsh Vegetation. *Functional Ecology* **3**, 363 (1989).

18. Wright, D. H. Species-Energy Theory: An Extension of Species-Area Theory. *Oikos* **41**, 496 (1983).

19. Fick, S. E. & Hijmans, R. J. WorldClim 2: new 1-km spatial resolution climate surfaces for global land areas: NEW CLIMATE SURFACES FOR GLOBAL LAND AREAS. *International Journal of Climatology* **37**, 4302–4315 (2017).

20. Sandel, B. *et al.* The Influence of Late Quaternary Climate-Change Velocity on Species Endemism. *Science* **334**, 660–664 (2011).

21. Lee, S.-M. & Chao, A. Estimating Population Size Via Sample Coverage for Closed Capture-Recapture Models. *Biometrics* **50**, 88 (1994).

22. Vavrek, M. J. Fossil: palaeoecological and palaeogeographical analysis tools. *Palaeontologia Electronica* **14**, (2011).

23. Hijmans, R. J., Philips, S., Leathwick, J. & Elith, J. Dismo: Species distribution modeling. R package version 1.0-12. (2017).

24. Barton, K. MuMin: Mutli-model inference. (2016).
